# Relationships of tumor differentiation and immune infiltration in gastric cancers revealed by single-cell RNA-seq analyses

**DOI:** 10.1007/s00018-023-04702-1

**Published:** 2023-02-02

**Authors:** Xin Zhou, Jingwei Yang, Yongqu Lu, Yanpeng Ma, Yan Meng, Qingqing Li, Junpeng Gao, Zhaoyu Jiang, Limei Guo, Wei Wang, Yun Liu, Lu Wen, Miao Kai, Wei Fu, Fuchou Tang

**Affiliations:** 1 School of Life Sciences, Biomedical Pioneering Innovation Center, Department of General Surgery, Third Hospital, Peking University, Beijing, 100871 People’s Republic of China; 2 Beijing Advanced Innovation Center for Genomics (ICG), Ministry of Education Key Laboratory of Cell Proliferation and Differentiation, Beijing, 100871 People’s Republic of China; 3 Academy for Advanced Interdisciplinary Studies, Peking University, Beijing, 100871 People’s Republic of China; 4 Peking-Tsinghua Center for Life Sciences, Peking University, Beijing, 100871 People’s Republic of China; 5 Peking University Third Hospital Cancer Center, Beijing, 100191 People’s Republic of China; 6 Faculty of Health Sciences, Cancer Center, University of Macau, Macau SAR, People’s Republic of China; 7 Department of Pathology, School of Basic Medical Science, Peking University Third Hospital, Peking University Health Science Center, Beijing, 100191 People’s Republic of China; 8 Department of Breast and Thyroid Surgery, China-Japan Friendship Hospital, Beijing, 100029 People’s Republic of China

**Keywords:** Intra-tumor heterogeneity, Pathological phenotype, Tumor immune microenvironment

## Abstract

Gastric cancers are highly heterogeneous malignant tumors. To reveal the relationship between differentiation status of cancer cells and tumor immune microenvironments in gastric cancer, single-cell RNA-sequencing was performed on normal mucosa tissue, differentiated gastric cancer (DGC) tissue, poorly differentiated gastric cancer (PDGC) tissue and neuroendocrine carcinoma (NEC) tissue sampled from surgically resected gastric cancer specimens. We identified the signature genes for both DGC and PDGC, and found that signature genes of PDGC strongly enriched in the epithelial–mesenchymal transition (EMT) program. Furthermore, we found that DGC tends to be immune-rich type whereas PDGC tends to be immune-poor type defined according to the density of tumor-infiltrating CD8^+^ T cells. Additionally, interferon alpha and gamma responding genes were specifically expressed in the immune-rich malignant cells compared with immune-poor malignant cells. Through analyzing the mixed adenoneuroendocrine carcinoma, we identified intermediate state malignant cells during the trans-differentiation process from DGC to NEC, which showed double-negative expressions of both DGC marker genes and NEC marker genes. Interferon-related pathways were gradually downregulated along the DGC to NEC trans-differentiation path, which was accompanied by reduced CD8^+^ cytotoxic T-cell infiltration. In summary, molecular features of both malignant cells and immune microenvironment cells of DGC, PDGC and NEC were systematically revealed, which may partially explain the strong tumor heterogeneities of gastric cancer. Especially along the DGC to NEC trans-differentiation path, immune-evasion was gradually enhanced with the decreasing activities of interferon pathway responses in malignant cells.

**Supplementary Information:**

The online version contains supplementary material available at 10.1007/s00018-023-04702-1.

## Introduction

Worldwide, gastric cancer ranks fifth in incidence and fourth in mortality, especially in Eastern Asia and Eastern Europe, where the incidence is significantly higher than that in other areas [1, 2]. Gastric cancer is a malignant entity of various pathological types, including well/moderately differentiated adenocarcinoma, poorly differentiated adenocarcinoma, mucous adenocarcinoma and signet ring cell carcinoma [3, 4]. In addition to the above “inter-tumor” heterogeneity, significant “intra-tumor” heterogeneity exists in gastric cancer [5, 6]. Clinically, it is common for different tumor components to coexist in the same gastric cancer, including differentiated adenocarcinoma, poorly differentiated adenocarcinoma or signet ring carcinoma [3, 7]. Some specific tumor components, such as hepatoid adenocarcinoma, neuroendocrine carcinoma and adenocarcinoma with enterocyte differentiation, make this heterogeneity more drastic and result in an even worse prognosis [8–12].

With the development of high-throughput sequencing technology, the understanding of the biological characteristics of gastric cancer has gradually deepened [13–16]. The Cancer Genome Atlas (TCGA) and Asian Cancer Research Group (ACRG) have defined specific molecular subtypes of gastric cancer, which to some extent has promoted progress in the clinical treatment of gastric cancer [17, 18]. Recent advances in single-cell sequencing techniques have led to further insights into the carcinogenesis and progression of gastric cancer [19–24], particularly the tumor microenvironment [25–29]. However, there is still a lack of research on the mechanisms of how tumor epithelial cells in different subtypes of gastric cancer affect the tumor immune microenvironment differently, and few studies have emphasized on the heterogeneity of gastric cancer. Here, utilizing a single-cell transcriptomic sequencing approach, we compared the differences between differentiated gastric cancer and other types of gastric cancer components, thus further elucidating the heterogeneity of gastric cancer and unveiling the relationship between gastric cancer cells and immune infiltration.

## Materials and methods

### Patients and samples

Normal mucosa was sampled at least 5 cm away from the tumor border. Samples were obtained immediately after surgical resection. Each sample was divided into 3 parts: one for single-cell RNA sequencing, one for pathological examination and one for cryopreservation. The histopathological information of the corresponding tissues was evaluated by two independent pathologists. Tumor staging was classified according to the 2017 TNM classification of the American Joint Committee on Cancer (AJCC).

### Single-cell cDNA amplification and library construction

We performed single-cell separation, DNA amplification and library construction following the manufacturer’s guidelines for chromium single-cell sequencing technology from 10X Genomics (USA). The sequencing libraries were constructed using the Chromium Single Cell 3ʹ Library and Gel Bead Kit V3. Finally, the prepared libraries were quality checked and sequenced on an Illumina NovaSeq 6000 platform.

### Laser capture microdissection

The tumor tissues were formalin-fixed and paraffin-embedded (FFPE) and then cut into 5-μm-thick sections, fixed with 95% ethanol and subjected to hematoxylin and eosin staining for conventional histological examination by three independent pathologists to determine the location of malignant cells. Then, experimental tissues (8 μm thick) that were placed onto membrane slides (MMI, Germany) for laser capture microdissection were fixed with 95% ethanol and stained with eosin. Samples on the slides were isolated by laser capture microdissection using the CellCut Plus system (MMI, Germany). The dissected tissues were lysed, and genomic DNA was extracted using the DNA Extraction Kit for FFPE samples (Amoy Diagnostics, China) according to the manufacturer’s protocol.

### Whole-exome sequencing (WES)

Extracted genomic DNA (approximately 200 ng) was fragmented by a Covaris system (Thermo Fisher Scientific, USA) into fragments of 150–200 bp. We used the DNA Clean & Concentrator Kit (Tianmo Biotech, China) for purification according to the manufacturer’s protocol. Next, libraries for WES capture were used with SureSelectXT Human All Exon V6 (Agilent Technologies, USA) following the provided instructions. The products were sequenced on an Illumina NovaSeq 6000 platform.

### Immune-histochemical staining

Tissues were cut into 5-μm-thick sections and were then deparaffinized and treated with 3% H_2_O_2_-CH_3_OH for 15 min to block endogenous peroxidase. The slices were submerged in pH 6.0 or 9.0 buffer for antigen retrieval and then incubated at 37 °C for 2 h with primary antibodies, including anti-CLDN3 (Abcam, ab214487, 1:500), anti-FABP1 (Abcam, ab171739, 1:4000), anti-PIGR (Abcam, ab275020, 1:500), anti-PHGR1 (SinoBiological, 204852-T08, 1:100), anti-MUC1 (Abcam, ab70475, 1:100), anti-CD8 (ZSGB-Bio, ZA-0508), anti-MUC6 (ZSGB-Bio, ZM-0396), anti-MUC5AC (ZSGB-Bio, ZA-0664), anti-Syn (ZSGB-Bio, ZM-0246) and anti-CgA (ZSGB-Bio, ZM-0076). After incubation with horseradish peroxidase (HRP)‐conjugated IgG (ZSGB‐Bio, China) at room temperature for 30 min, the cells were stained with a 3,3N-diaminobenzidine tetrahydrochloride (DAB) detection kit (ZSGB‐Bio, China). The expression and localization of proteins were detected under light microscopy.

### Processing of single-cell RNA-seq data

We processed our data following the manufacturer’s instructions of 10X Cell Ranger (version 3.1.0) with default arguments used to process the raw data, and we used the human GRCh38 genome as the reference genome. We retained cells with more than 500 detected genes, more than 1000 transcripts and less than 50% mitochondrial genes because of the high percentages of mitochondrial genes in parts of normal gastric cells. The unique molecular identifiers (UMIs) of each cell were normalized and transformed through log-transformation. The log-normalized values were used in the downstream analyses.

### Clustering and differentially expressed gene analysis

The R package Seurat (version 3.2.2) was used to perform the downstream analyses, and Harmony (version 1.0) was used to correct batch effects of different experimental batches [30, 31]. Individual cells were clustered through a graph-based clustering approach of Seurat. Differentially expressed genes (DEGs) of different cell types were identified through the FindAllMarkers function of Seurat using the Wilcoxon test and the fold change with a value of 2. The detailed parameters were as follows: test.use = 'wilcox', min.pct = 0.25, and logfc.threshold = log(1.5). Metascape (http://metascape.org/) was used to perform gene enrichment analysis [32].

### CNVs inferred by single-cell RNA-seq data

The R package CopyKAT (version 1.0.4) was used to infer CNVs based on the gene expression of all the epithelial cells in our single-cell RNA data [33]. The parameters ngene.chr = 5, win.size = 25, and KS.cut = 0.05 were used, and epithelial cells from the adjacent normal tissues were assigned as normal epithelial cells during the analysis. According to CNV outputs inferred by gene expression, the mean of squares of deviation as the measurement represents the CNV levels of single cells.

### Classification of malignant cells and non-malignant epithelial cells

**Fig. 1:**
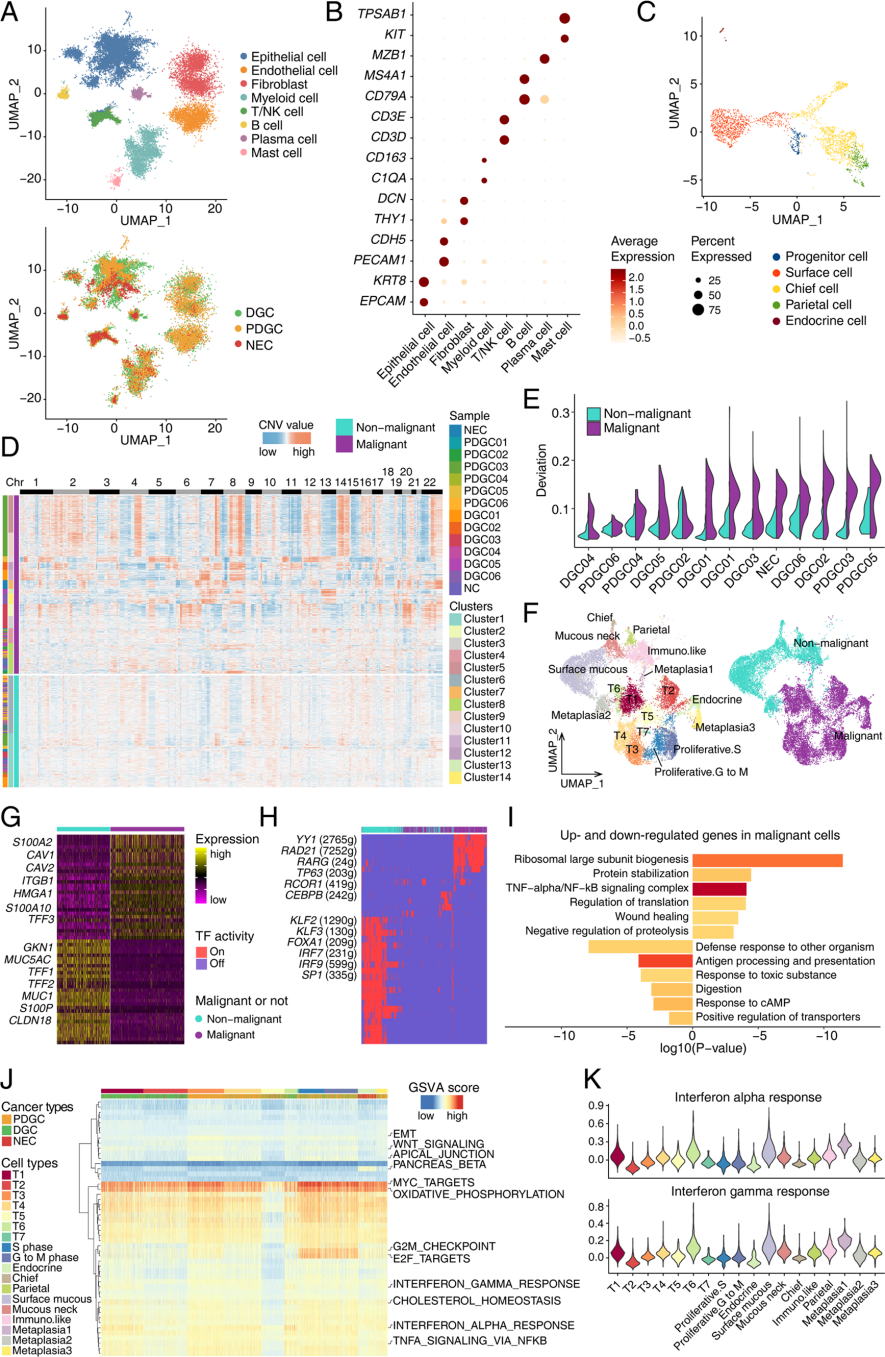
Representative single-cell transcriptome landscape of gastric cancer. **A** UMAP plot showing different cell types (*n* = 46,883), with pathological information including differentiated gastric cancer (DGC), poorly differentiated gastric cancer (PDGC) and neuroendocrine carcinoma (NEC). **B** The expression of corresponding markers for different cell types. **C** UMAP plot for one normal mucosa tissue (*n* = 2232). **D** Heatmap showing chromosomal landscape of large-scale copy number variations (CNV) inferred from single cell RNA data. **E** Violin plot showing CNV deviation of non-malignant and malignant epithelial cells from each case.** F** UMAP plot showing different clusters of epithelial cells. **G** Heatmap showing differentially expressed genes (DEGs) between malignant and non-malignant epithelial cells. Part gene names are displayed and the full gene list is in Supplementary Table S4. **H** Gene regulatory networks (GRNs) identified in malignant and non-malignant epithelial cells. Part transcription factor names are displayed. **I** Gene enrichment analysis showing the signaling pathways relatively up- or downregulated in malignant compared with non-malignant epithelial cells. **J** Heatmap showing the expressing scores of hallmark gene sets in malignant and non-malignant epithelial cells.** K** Violin plot showing the expression levels of interferon alpha and gamma response genes in different epithelial clusters

With the identified clusters of epithelial cells, cell clusters of epithelial cells with high CNV levels were manually annotated as malignant cells, and other clusters of epithelial cells with low CNV levels were annotated as non-malignant cells. The classification strategy is as following. First, we confirmed the CNV patterns of cancer cells by WES data from the corresponding laser capture microdissected tissues, which guaranteed a high cancer cell purity by combining pathology information (Fig. S2A). Second, we combined both clustering and CNV information to distinguish malignant cells from non-malignant epithelial cells. Non-malignant epithelial cells clustered together with normal epithelial cells from normal mucosa. And malignant cells were separated from non-malignant epithelial cells in tumor samples and epithelial cells from normal mucosa clearly (Fig. 1F and Fig. S2B). Third, non-malignant epithelial cells from different tumor samples clustered together with normal epithelial cells and mixed very well, while malignant cells were separated among tumor samples from different patients (Fig. S1G). The above information confirms the validity of our strategy to better distinguish malignant cells from non-malignant epithelial cells in tumor samples.

### CNVs inferred by WES data

For the WES data, low-quality and adapter-contaminated reads were removed and trimmed by Trimmomatic (version 0.39), and then clean reads were aligned to the human GRCh38 genome using BWA (version 0.7.17). Control-freec (version 11.5) was used to estimate CNVs, outputting the copy numbers of 10 M segments.

### Identification of the tumor differentiation gene set

DEGs of differentiated gastric cancer (DGC) compared with poorly differentiated gastric cancer (PDGC) were identified with the FindMarker function built in the Seurat package. Genes highly correlated with the DEGs of DGC were selected as candidate genes, with a Pearson correlation greater than 0.5. Among these candidate genes, we used the lasso regression and random forest methods to remove the uncertain genes and identify the tumor differentiation gene set. The glmnet function of the package glmnet (version 2.0–16) was applied to perform lasso regression, with the parameters alpha = 1 and family = 'binomial'. The randomForest function of the randomForest package (version 4.6–15) was used to perform random forest, with the parameters ntree = 1500 and mtry = 10. With prior knowledge about tumor differentiation, genes with low contributions assessed by lasso regression and random forest and with little association with tumor differentiation were removed from the candidate gene set, including *TM4SF20*, *HEPH*, *LINC01133*, *PPP1R1B*, *AKR1C3* and *REEP6*. Finally, we retained genes whose expressing level (the log-normalized value) is higher than the ln value of 0.5. The retained genes were identified as the tumor differentiation gene set and was shown in Supplementary Table S6.

### Identification of gene expression patterns in gastric cancer cells

To identify diverse gene expression programs of malignant epithelial cells, we first used the AUCell package (version 1.6.1) to score malignant cells in each sample for highly variable genes and carried out feature extraction using nonnegative matrix factorization (NMF) with the default “scd” decomposition algorithm and 10 factors in the factorization per tumor sample with the function implemented in the NNLM package (version 0.4.2). Then, the Pearson correlation coefficients of factors from different tumor samples were clustered and manually divided into several programs. These programs were annotated with specific functional genes and pathways.

### Gene regulatory network (GRN) analysis

The R package SCENIC (version 1.1.3) was used to assess the transcription factor strength [34]. We used the GENIE3 method to detect correlations based on the dataset of motifs located 20 kb around TSSs from the cisTarget database.

### Gene set signature and signaling pathway enrichment analysis

To assess the expression levels of different signatures at the single-cell resolution, the AddModuleScore function of Seurat was used to score gene sets of single cells. We also used the Gene Set Variation Analysis (GSVA) enrichment scores to describe enrichment scores of the hallmark gene sets at the single-cell levels through the R package GSVA (version 1.32.0). The “ssgsea” method of the GSVA package was used [35].

### Quantifying metabolic activity at single-cell resolution

The R package scMetabolism (version 0.2.1) was used to quantify different types of metabolic activities at the single-cell level [36]. We used the parameters of method = 'VISION' and metabolism.type = 'KEGG' to perform the analysis.

### Pseudotime analysis

The R package Monocle2 (version 2.12.0) with the DDR-Tree method and default parameters was used to perform the single-cell trajectory analysis [37]. We used the DEGs or marker genes of individual clusters as ordering gene sets. The cell trajectories were inferred with the default parameters after dimension reduction and cell ordering.

### Cell communication analysis

The toolkit NATMI was used to perform cell communication analysis [38]. The ExtractEdges function calculates the expression and specificity of ligand–receptor interactions, and the DiffEdges function identifies changes in ligand–receptor interactions between two conditions. The built-in “lrc2p” database was used to predict interactions, the weight of edges was calculated by the mean method, and the detection threshold value was set to 0.2.

### Survival analysis

Survival analysis of gastric cancer samples from the TCGA dataset based on the expression status of identified genes was carried out by the survival package (version 0.4.8) and the survminer package (2.44–1). The assumption of the Cox proportional hazards model was tested using cox with 0.1 as the cut-off value, and the Cox proportional hazards model was fit using patient groups divided by the median gene expression level.

## Results

### Representative single-cell transcriptome landscape of gastric cancer obtained by a pathology-informed sampling strategy

We applied a pathology-informed sampling strategy and droplet-based scRNA-seq (10X Genomics) on 14 tissues sampled from surgically resected gastric cancer specimens, including 1 normal mucosa tissue, 6 differentiated gastric cancer (DGC) tissues, 6 poorly differentiated gastric cancer (PDGC) tissues and 1 neuroendocrine carcinoma (NEC) tissue, all of which were confirmed by side-by-side histopathological examination (Supplementary Table S1). The neuroendocrine carcinoma tissue and a corresponding adenocarcinoma tissue were from the same tumor pathologically diagnosed as mixed adenoneuroendocrine carcinoma (MANEC).

After stringent quality control, 46,883 cells were ultimately retained for further analyses, which detected a median of 1723 genes and 5480 unique molecular identifiers (UMIs) per cell (Fig. S1A). After dimension reduction and clustering, 8 clusters of cells were identified, and the captured cells were clearly divided into epithelial cells (*EPCAM* and *KRT8*), fibroblasts (*THY1* and *DCN*), endothelial cells (*PECAM1* and *CDH5*), myeloid cells (*C1QA* and *CD163*), T/NK cells (*CD3D* and *CD3E*), B cells (*CD79A* and *MS4A1*), plasma cells (*MZB1*) and mast cells (*KIT* and *TPSAB1*) according to the expression of well-known marker genes (Supplementary Table S2 and Fig. 1A, B). The percentages of these cell types differed in tumor samples, and epithelial cells and T/NK cells comprised the majority (Fig. S1B–E).

We captured one normal mucosa tissue in our data, and epithelial cells of this sample were divided into progenitor cells, surface cells, chief cells, parietal cells and endocrine cells according to well-known markers (Fig. 1C and Fig. S1F). Then, we estimated CNV levels of epithelial cells from tumor tissues, with high-CNV and low-CNV epithelial cells existed in every tumor sample (Fig. 1D, E). Cells with high CNVs were confirmed by WES data from the corresponding laser capture microdissected tissue (Fig. S2A). To classify malignant cells from non-malignant epithelial cells in our data, we combined clustering and CNV information to distinguish malignant cells from non-malignant cells (Fig. S1G, H), resulting in 11 malignant cell clusters and 7 non-malignant cell clusters (Supplementary Table S3, Fig. 1F and Fig. S1I, J). Non-malignant epithelial cells in tumor samples from different patients clustered together with the epithelial cells from normal mucosa, while malignant cells were clustered by samples and separated from non-malignant epithelial cell clusters (Fig. S2B, C). By comparing malignant and non-malignant epithelial cells, we found that malignant cells lost digestive and defense functions but enhanced protein synthesis activities and metabolism (Supplementary Table S4 and Fig. 1G, I). We also assessed the gene regulatory networks (GRNs) of malignant and non-malignant cells and clustered them based on GRNs that classified epithelial cells according to different patients (Fig. S2D). GRNs, such as those for *YY1*, *RAD21*, *RARG*, *TP63* and *CERBPB,* were identified in malignant cells, while several GRNs, including those for *SP1*, *IRF7*, *IRF9*, *FOXA1* and *KLF2,* were identified in non-malignant epithelial cells (Fig. 1H).

We assessed different signatures of malignant clusters and found that endocrine cells represented some pancreatic beta cell-like signatures, which displayed secretion-related features (Fig. 1J and Fig. S2G). Moreover, the T1 and T6 clusters represented interferon alpha and gamma response signatures, which implies that the T1 and T6 clusters showed unique immunological related features (Fig. 1J, K and Fig. S2E, F). We also evaluated the metabolism-related activities in epithelial clusters and found that glycerolipid and glycerophospholipid metabolism activities were higher in T6 than in other malignant clusters (Fig. S2H, I).

### Differentiation signature of cancer cells identified by comparing DGC with PDGC

**Fig. 2:**
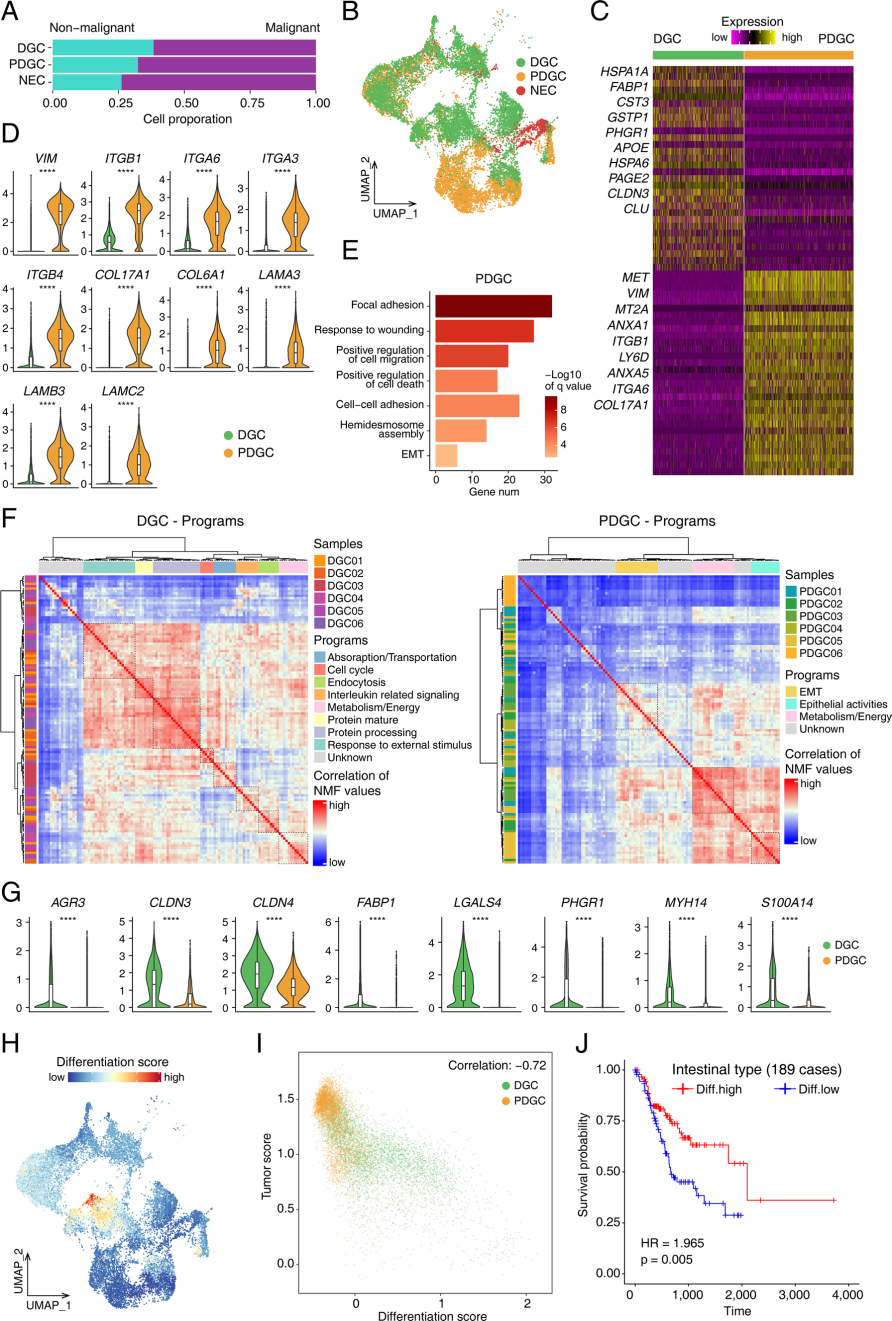
Comparison between differentiated gastric cancer (DGC) and poorly differentiated gastric cancer (PDGC). **A** The percentage of malignant cells in DGC, PDGC and neuroendocrine carcinoma (NEC). **B** UMAP plot showing distribution of epithelial cells from DGC, PDGC and NEC. **C** Heatmap showing differentially expressed genes between malignant epithelial cells from DGC and PDGC. Part gene names are displayed and the full gene list is in Supplementary Table S5. **D** Violin plot showing the expression levels of EMT-associated genes in DGC and PDGC. **E** Gene enrichment analysis showing the functions relatively upregulated in PDGC. **F** Non-negative matrix factorization (NMF) showing transcriptomic programs featured by DGC and PDGC. **G** Violin plot showing the expression levels of genes involved in differentiation score from DGC and PDGC (****—*p* value < 0.0001 calculated by wilcox test). **H** UMAP plot showing the levels of differentiation score. **I** The correlation between the tumor differentiation score and tumor score. **J** Kaplan–Meier survival (days) curve showing the survival of intestinal-type gastric cancer cases in the TCGA dataset with high and low differentiation score

We compared the transcriptomes of DGCs and PDGCs with a clear pathological diagnosis to clarify their molecular differences (Fig. 2A, B). As for DEGs of malignant cells from PDGC and DGC, the expressions of *CLDN3*, *FABP1*, *S100A10* and *PHGR1* were significantly higher in DGCs, while epithelial–mesenchymal transition (EMT)-related genes such as *VIM*, *LAMB3*, *LAMC2*, *COL6A1* and *COL17A1* were specifically expressed in PDGCs¸ indicating that the EMT signature was enriched in PDGCs (Supplementary Table S5, Fig. 2C, D and Fig. S3A). Several EMT-related terms such as focal adhesion, response to wounding, positive regulation of cell migration and cell–cell adhesion were enriched in PDGC (Fig. 2E). Compared with non-malignant epithelial cells, there were more upregulated and downregulated DEGs in PDGCs than in DGCs (Fig. S3B, S3D). In addition, *HIF1A* was mainly expressed in PDGCs, indicating potentially stronger hypoxia in PDGCs (Fig. S3E).

To assess the gene expression programs of DGCs and PDGCs, we used the nonnegative matrix factorization (NMF) method to analyze malignant cells of different cancer types. We found that the EMT, epithelial-related and metabolism/energy programs were enriched in PDGCs which was confirmed by gene enrichment analysis, whereas absorption/transportation, cell cycle, endocytosis, interleukin-related signaling, metabolism/energy, protein maturation, protein processing and response to external stimulus programs were enriched in DGCs (Fig. 2F).

Based on upregulated genes highly correlated with malignant cells of DGCs, we used the lasso regression and random forest methods to trim low-contribution genes. Finally, we identified a gene set associated with the differentiation status of gastric cancer, including *AGR3*, *CLDN3*, *CLDN4*, *FABP1*, *LGALS4*, *PHGR1*, *MYH14* and *S100A14* (Supplementary Table S6 and Fig. S3F, G). The identified tumor differentiation gene set had significantly higher expression in DGCs than in PDGCs (Fig. 2G and Fig. S3H). This gene signature clearly defined DGC, especially in the T1 and T6 clusters in our dataset (Fig. 2H). Additionally, we proposed a score based on the upregulated markers of all malignant cells as the tumor score. The tumor score had a negative correlation with our identified tumor differentiation gene set, which indicated that a higher differentiation level of cancer cells represented a less malignant signature of the tumor (Fig. 2I). Moreover, in the TCGA database, the higher expression of our differentiation signature gene set was clearly associated with better overall survival in intestinal-type gastric cancer (Fig. 2J).

### Differentiation signatures partially overlapped with immune infiltration-related genes

**Fig. 3:**
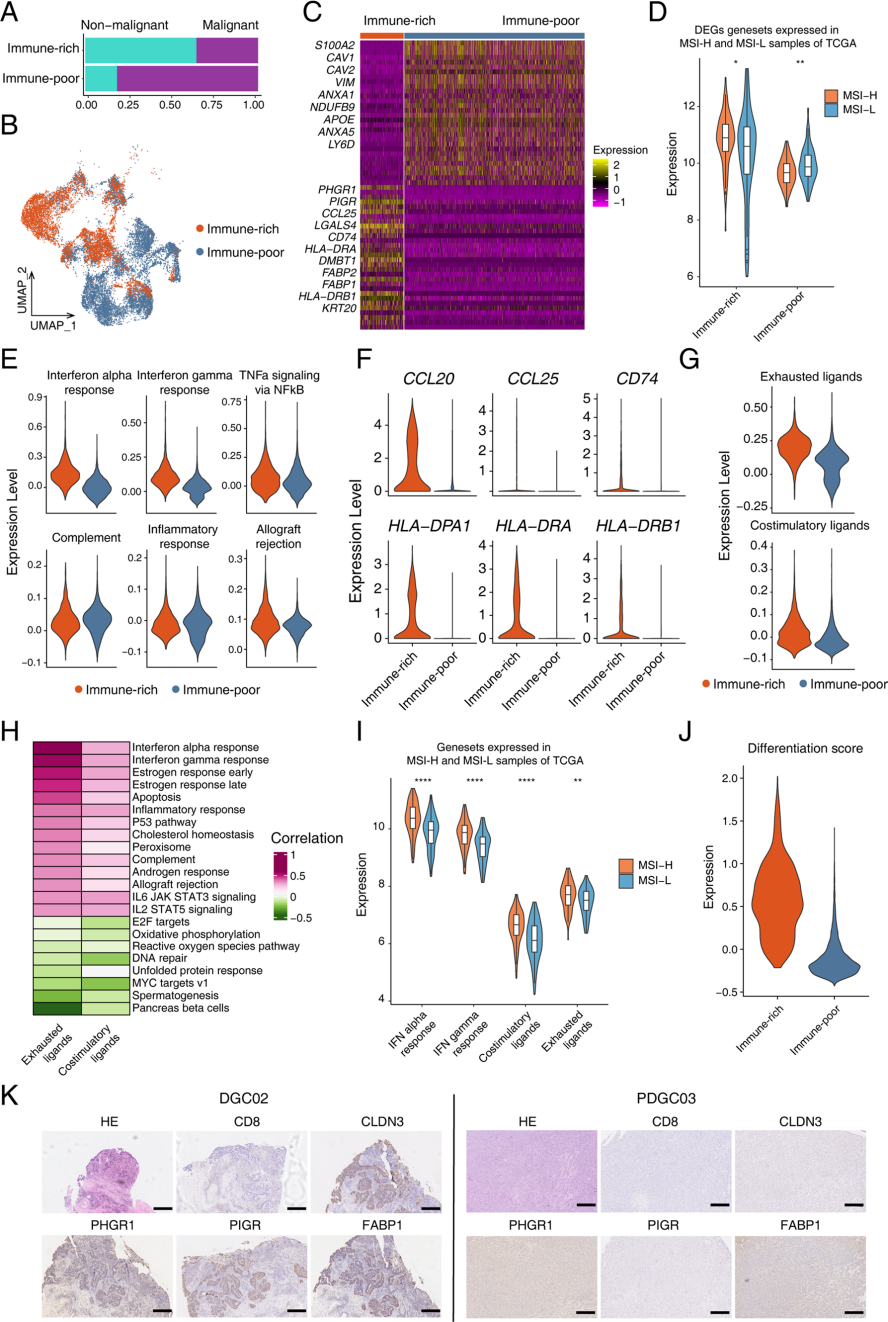
Transcriptomic profiling of immune infiltration-related genes. **A** The percentage of malignant cells in immune-rich and immune-poor type samples. **B** UMAP plot showing distribution of epithelial cells from immune-rich and immune-poor type samples. **C** Heatmap showing differentially expressed genes between immune-rich and immune-poor type gastric cancer. Part gene names are displayed and the full gene list is in Supplementary Table S7. **D** Violin plot showing expression levels of markers for immune-rich malignant cells in microsatellite instability-high (MSI-H) and microsatellite instability-low (MSI-L) samples from the TCGA dataset (ns—*P* value >  = 0.05, *—*P* value < 0.05, **—*P* value < 0.01, ***—*P* value < 0.001, ****—*P* value < 0.0001 calculated by *t* test). **E** Violin plot showing expression levels of hallmark signatures and GO gene sets of immune response in immune-rich and immune-poor type gastric cancer. **F** Violin plot showing expression levels of genes in response to interferon gamma and antigen presentation-related genes in immune-rich and immune-poor type gastric cancer. **G** Violin plot showing expression levels of exhausted ligand-related and costimulatory ligand-related genes in immune-rich and immune-poor type gastric cancer. **H** Hallmark gene signatures highly correlated with exhausted ligand and costimulatory ligand gene sets. **I** Violin plot showing expression levels of interferon alpha/gamma response genes, exhausted ligand and costimulatory ligand-related response genes in MSI-H gastric cancer samples in MSI-H and MSI-L samples from the TCGA dataset (ns—*P* value >  = 0.05, *—*P* value < 0.05, **—*P* value < 0.01, ***—*P* value < 0.001, ****—*P* value < 0.0001 calculated by t test). **J** Violin plot showing differentiation score in immune-rich and immune-poor type gastric cancer. **K** Representative images of hematoxylin–eosin staining, and immune-histochemical staining of CD8 and other differentially expressed proteins in immune-rich and immune-poor type samples. Scale bar, 300 μm

Next, to study the relationship between the differentiation signature of the cancer cells and characteristics of the corresponding immune microenvironments, we divided the patients into the immune-poor type and immune-rich type according to the density of tumor-infiltrating CD8^+^ T cells [39]. We found that the proportion of malignant cells was higher in the immune-poor type, which indicated the high density of malignant cells in immune-poor tumors, as indicated by H&E staining (Fig. 3A, B). We identified markers such as *S100A2*, *CAV1*, *CAV2* and *ANXA1* and GO terms including focal adhesion, integrin-related pathways and response to wounding in the immune-poor type, while genes such as *LAGLS4*, *CLDN3* and *PIGR* and GO terms including granulocyte migration, neutrophil degradation and endocytosis were identified in the immune-rich type (Supplementary Table S7, Fig. 3C and Fig. S4A). Moreover, gastric cancer samples of MSI-H and MSI-L in the TCGA dataset were used to confirm DEGs between immune-poor and immune-rich types, and as expected, markers of malignant cells in the immune-poor type tumor tissues had a higher expression in MSI-L samples, while markers of malignant cells in the immune-rich type tumor tissues had a higher expression in MSI-H samples in the TCGA dataset (Fig. 3D). The percentage of malignant cells from immune-rich and immune-poor types differed in malignant clusters, and malignant cells in the immune-rich type tumor tissues were enriched in the T1, T6 and T7 clusters (Fig. S4C). Moreover, we analyzed the pseudotime trajectory of malignant cells according to the immune-related gene sets, and the path of malignant cells from immune-rich types to immune-poor types was identified, indicating a continuous transition between these two types (Fig. S4B).

We utilized several hallmark signatures and GO gene sets of immune responses to score tumor cells from immune-rich and immune-poor types. The cancer cells in immune-rich type tumors tended to have higher expression of interferon alpha and interferon gamma response genes (Fig. 3E and Fig. S4D). Genes in response to interferon gamma, including *CCL20*, *CCL25* and *CD74,* and antigen presentation-related genes, such as *HLA-DPA1*, *HLA-DRA* and *HLA-DRB,* were significantly upregulated in the cancer cells of immune-rich type tumors (Fig. 3F). Moreover, to identify the responses of tumor cells to infiltrative T cells, we studied the exhausted and costimulatory receptors and found that the cancer cells of immune-rich type tumors showed higher expression of both the corresponding exhausted ligand-related and costimulatory ligand-related genes compared with the immune-poor type (Fig. 3G). Meanwhile, the hallmark gene signatures highly correlated with exhausted ligand and costimulatory ligand gene sets were enriched in immune-related activities, such as interferon gamma response, interferon alpha response, inflammatory responses and complement activities (Fig. 3H). We also screened these features in the TCGA dataset and found higher expression of interferon alpha/gamma response genes, exhausted ligand and costimulatory ligand-related response genes in MSI-H gastric cancer samples, which were characterized by heavy immune infiltration (Fig. 3I).

As seen from the heatmap, the DEGs between the immune-poor type and the immune-rich type in general overlapped with those between PDGC and DGC, and the malignant cells in the immune-rich type had a higher differentiation score than those in the immune-poor type (Fig. 3J, K). We also identified genes that were highly correlated with interferon gamma and the interferon alpha pathway, and several genes were also differentially expressed in the malignant cells between the immune-rich and immune-poor types (Fig. S4E). For example, *PHGR*1 and *CLDN3* showed significantly higher expression in the malignant cells of the immune-rich type and was mainly expressed in the T1 and T6 clusters (Fig. S4F). Meanwhile, their expression was associated with better prognosis of intestinal-type gastric cancer in the TCGA database (Fig. S4G). Finally, we also identified metabolic activities highly correlated with interferon alpha/gamma signatures and found that lipid-related signatures, such as retinol metabolism and steroid hormone biosynthesis, were positively correlated with the immune-rich type (Fig. S4H, I), while pyruvate-related signatures, such as purine metabolism, pyruvate metabolism and pyrimidine metabolism, were negatively correlated with the immune-rich type [40].

### Tumor infiltrating T-cell characteristics and their interactions with malignant cells

**Fig. 4:**
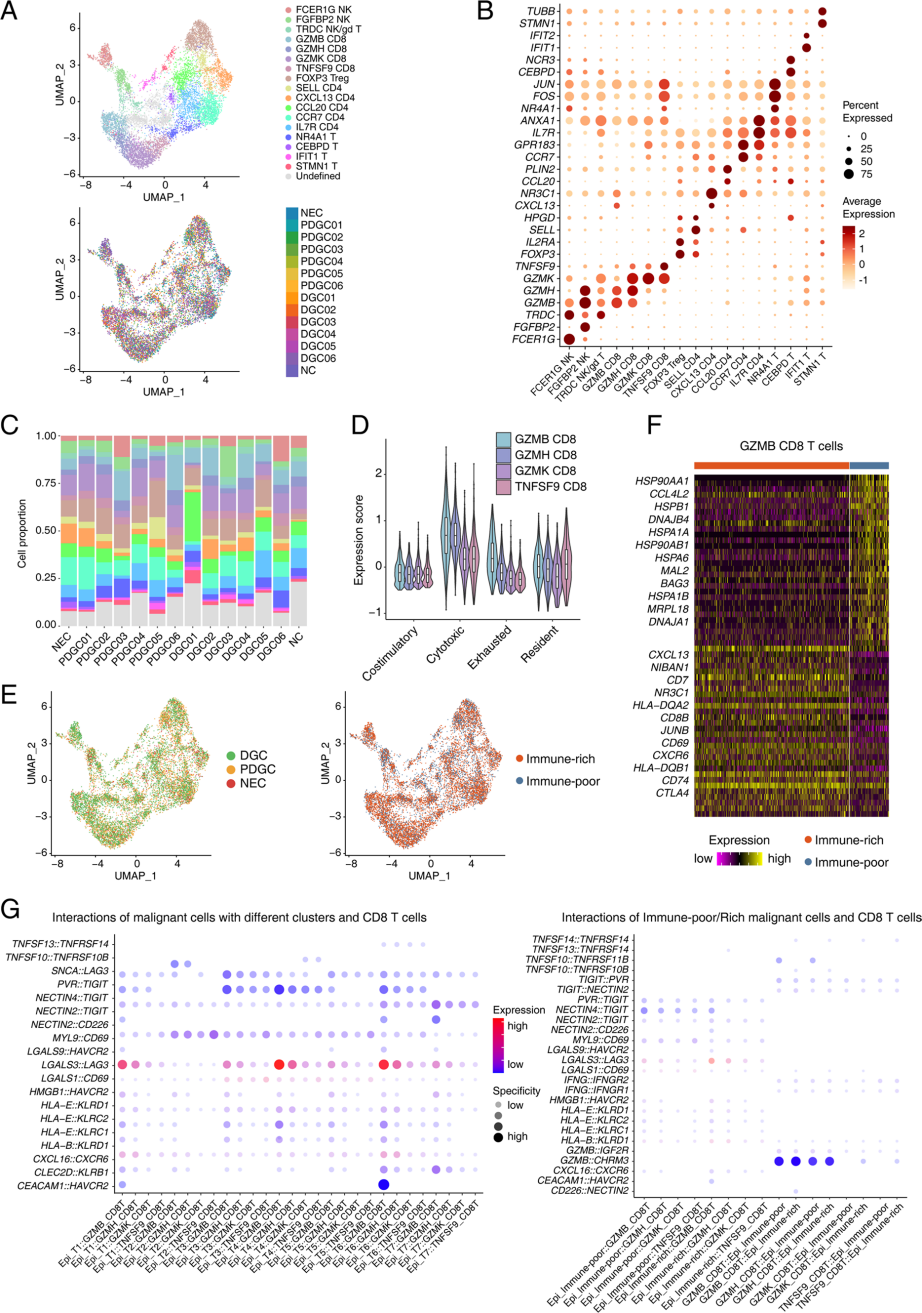
Tumor infiltrating T cell characteristics and interactions with malignant cells. **A** UMAP plot showing different clusters of T cells. **B** The expression of corresponding markers for different T cell clusters. **C** The percentage of different types of T cells in each sample. **D** Costimulatory, cytotoxic, exhausted and resident T cell signature score in different types of CD8^+^ T cells. **E** UMAP plot showing distribution of T cells across PDGCs and DGCs, immune-rich and immune-poor types. **F** Heatmap showing differentially expressed genes between *GZMB*^+^ CD8^+^ T cells from immune-rich and immune-poor type gastric cancer. Part gene names are displayed. **G** Interactions between epithelial cells and CD8^+^ T cells inferred from cell communication analysis using the toolkit NATMI

To delineate NK/T-cell clusters and the association between T cells and malignant cells in detail, we analyzed all the T cells and divided them into 17 clusters based on the corresponding markers, including 3 NK or γ/δ T-cell clusters, 4 CD8^+^ T-cell clusters, 6 CD4^+^ T-cell clusters and 4 other T-cell clusters of specific signatures (Fig. 4A, B). These clusters were distributed relatively evenly in different tumor samples (Fig. 4C). Of these T-cell clusters, STMN1^+^ T cells represent the cell cycle signature and contain both CD8^+^ and CD4^+^ T cells (Fig. S5A). For these four CD8^+^ T cell clusters (GZMB^+^ CD8^+^ T cells, GZMH^+^ CD8^+^ T cells, GZMK^+^ CD8^+^ T cells and TNFSF9 CD8^+^ T cells), GZMB^+^ CD8^+^ T cells and GZMH^+^ CD8^+^ T cells have a stronger cytotoxic ability than others, while GZMB^+^ CD8^+^ T cells have a specific exhausted signature compared with other CD8^+^ T cells (Fig. 4D). We screened out exhaustion-related genes such as *PDCD1*, *CTLA4* and *HAVCR*, which are mainly expressed in GZMB^+^ CD8^+^ T cells (Fig. S5B).

T cells from both DGCs and the immune-rich type account for the majority of T cells we captured (Fig. 4E). Moreover, all of the four main functional CD8^+^ T cell types were significantly enriched in the immune-rich type, which was consistent with the pathological findings (Fig. S5C, D). We found that GZMB^+^ CD8^+^ T cells from the immune-rich type had a higher expression of *CXCL13* (Fig. 4F and Fig. S5E), which was also reported to be associated with good responses to immunotherapies in triple-negative breast cancer [41]. Also, we found that energy-producing functions such as oxidative phosphorylation, glycolysis/gluconeogenesis and the citrate cycle had a positive correlation with cytotoxic and exhausted signatures (Fig. S5F, G), indicating that the cytotoxic and exhausted functions of CD8^+^ T cells probably consume more energy.

Then we conducted an in-depth analysis of cell–cell communications. For different clusters of malignant cells and CD8^+^ T cells, we found that exhaustion-related ligands were mainly enriched in the T1, T4 and T6 malignant clusters, and exhaustion-related receptors were mainly enriched in GZMB^+^ CD8^+^ T cells, especially interactions such as *LAGLS3-LAG3*, *CEACAM1-HAVCR2* and *NECTIN4-TIGIT*, which indicated that these pairs of malignant cells and CD8^+^ T cells were involved in T-cell exhaustion (Fig. 4G). Regarding the communications between immune types and different types of CD8^+^ T cells, we also identified exhaustion-related interactions between malignant cells and GZMB^+^ CD8^+^ T cells, while the corresponding ligands were distributed in both immune-poor and immune-rich malignant cells, especially interactions such as *LAGLS3*-*LAG3* and *HLA-B*-*KLRD1* (Fig. 4G). Moreover, we identified interactions between malignant cells and CD4^+^ T cells. Interactions such as *NECTIN4*-*TIGIT* and *MYL9*-*CD69* were distributed in almost all the interaction pairs of different types of malignant cells and CD4^+^ T cells, but they were more specific in CXCL13^+^ CD4^+^ T cells (Fig. S5H).

### Transition of differentiation status in mixed adenoneuroendocrine carcinoma

**Fig. 5:**
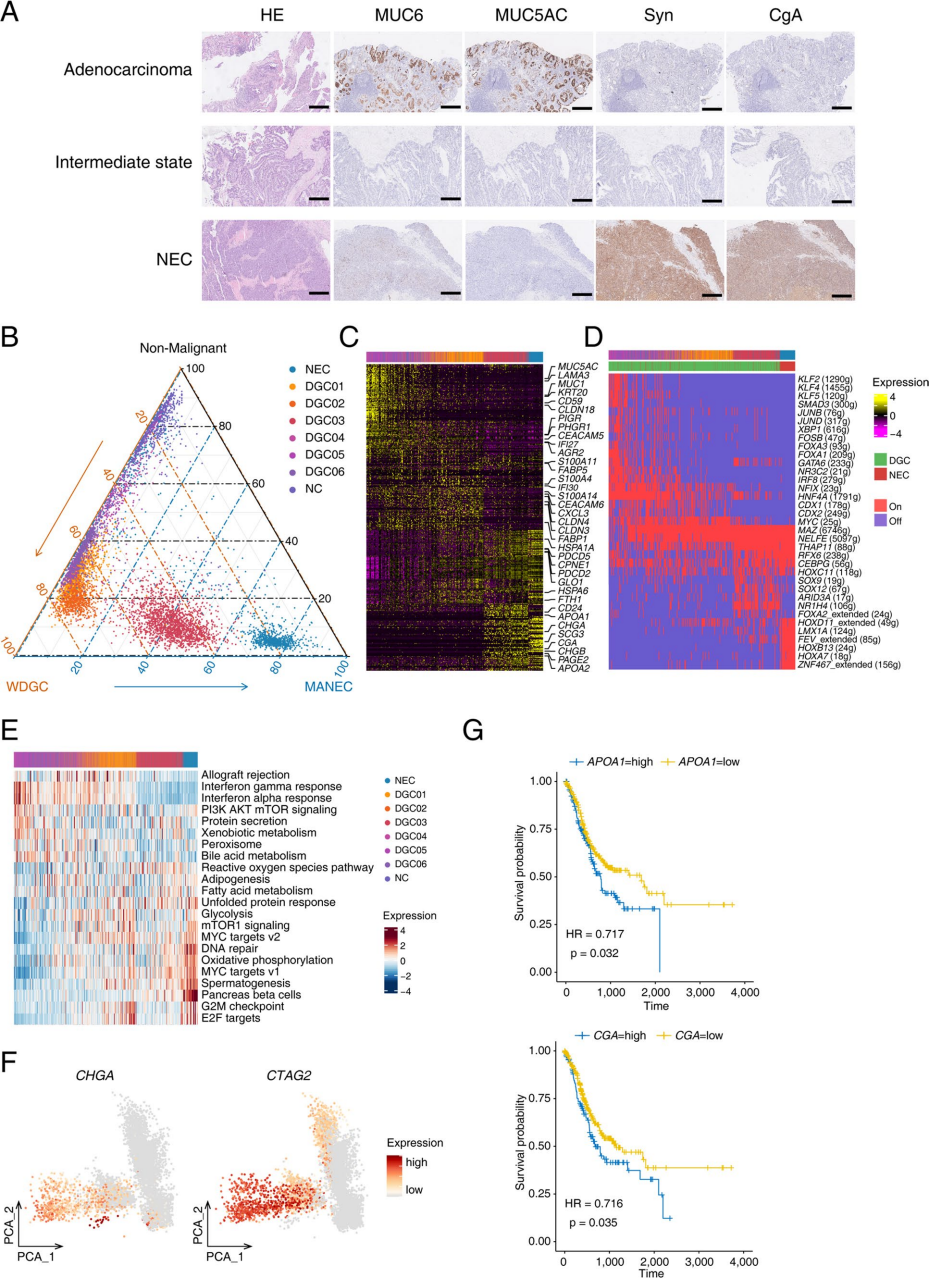
Transition of differentiation status in mixed adenoneuroendocrine carcinoma. **A** Representative images of hematoxylin–eosin staining, and immune-histochemical staining of MUC6, MUC5AC, Syn and CgA in adenocarcinoma, intermediate state and neuroendocrine carcinoma (NEC) components from the same mixed adenoneuroendocrine carcinoma. Scale bar, 300 μm. **B** Pseudotime trajectory of all the epithelial cells from non-malignant epithelial cells, malignant cells from differentiated gastric adenocarcinoma (DGC) to NEC. **C** Heatmap showing genes that changed gradually along the trans-differentiation path of DGC to NEC. Part gene names are displayed. **D** Gene regulatory networks identified in malignant cells of NEC and DGC. Part TF names are displayed. **E** Heatmap showing the expressing scores of hallmark gene sets along the trans-differentiation path of DGC to NEC. **F** PCA plot showing the expression of *CHGA* and *CTAG2* in non-malignant epithelial cells, and epithelial cells from DGC and NEC. **G** Kaplan–Meier survival curve showing the survival (days) of gastric cancer cases in the TCGA dataset with high and low expression levels of *APOA1* and *CGA*

Of these 12 patients we sequenced, 2 had MANEC, which was defined as a type of gastric cancer with the morphological and immunophenotypic characteristics of both classic adenocarcinoma and neuroendocrine carcinoma. Combining morphological and immune-histochemical analysis, we identified three prevalent components in these 2 MANEC tumors: differentiated adenocarcinoma, intermediate state cancer cells and neuroendocrine carcinoma. The intermediate state cancer cells referred to tumor components that were morphologically intermediate between differentiated adenocarcinoma and neuroendocrine carcinoma but did not express the well-known markers of either differentiated adenocarcinoma (MUC5AC and MUC6) or neuroendocrine carcinoma (Syn and CgA), although it can be regarded as differentiated adenocarcinoma in pathological examination (Fig. 5A).

Since both patients had differentiated gastric adenocarcinoma with neuroendocrine carcinoma components, we combined single-cell transcriptome data from all differentiated gastric cancer, intermediate state and neuroendocrine carcinoma components to explore the origin of neuroendocrine components. DEGs and differentially expressed TFs between DGCs and NECs were analyzed in our dataset, and endocrine-related signatures and genes specific to NECs, such as *CGA*, *CHGA*, and *CHGB,* as well as NEC specific TFs, such as *HMGN3*, *HOXD11* and *FEV,* were identified (Fig. S6D). The PCA reduction of all the epithelial cells showed a gradual change from differentiated gastric adenocarcinoma to an intermediate state and then to neuroendocrine carcinoma (Fig. S6A). Based on PCA components, we created the pseudotime path of all the epithelial cells from non-malignant epithelial cells, DGCs to NECs, with three poles of epithelial cells and different signatures identified (Fig. 5B). We identified genes that changed gradually along the trans-differentiation path. *KRT20*, *PHGR1*, *PIGR* and *CLDN3* were gradually downregulated, while *PDCD5*, *CPNE1*, *PDCD2*, *GLO1*, *APOA1* and *CGA* were upregulated (Fig. 5C and Fig. S6B), indicating that the differentiation score gradually decreased as the expression of the neuroendocrine markers increased. Differentiation-related TFs such as *KLF2*, *KLF3* and *FOXA3* were downregulated, and endocrine-related TFs such as *HOXA7*, *FEV* and *LMX1A* were upregulated along the trans-differentiation path (Fig. 5D). Especially, potential targets of immunotherapy including *MUC1*, *CEACAM5* and *CLDN18* were gradually downregulated along the trans-differentiation path*.* On the contrary, *CTAG2*, a cancer-testis antigen, was gradually upregulated toward NEC status (Fig. 5F). Moreover, the expression of *APOA1* and *CGA* was associated with poorer prognosis in gastric cancer from the TCGA dataset, and *CGA* is a typical marker of NECs (Fig. 5G). We noticed that malignant cells of the intermediate state expressed neither the well-known DGC signature genes (*MUC1, KRT20*, *PHGR1*) nor the NEC signature genes (*SCG3*, *CGA*, *CHGB*), which was consistent with the aforementioned morphological and immune-histochemical staining findings. Using the hallmark gene set, we can see the changing signatures along the trans-differentiation path, and signatures such as unfolded protein response, fatty acid metabolism, glycolysis and MYC targets were specifically enriched in the intermediate state (Fig. 5E). Several genes including *LGALS1, DDIT4* and *EIF4G1* were also enriched in the intermediate state (Fig. S6C).

The negative correlation of interferon-related pathways was also found along the DGC to NEC trans-differentiation path (Fig. 5E and Fig. S6E, F). The decrease of immune responding signatures in malignant cells was accompanied by reduced CD8^+^ T cell infiltration in IHC staining (Fig. S6B). Analysis of specific genes in interferon-related pathways also confirmed a similar correlation, such as the expression of *CASP1*, *CD74*, *B2M*, *IFITM3* and *IFI27,* which have negative correlations with NEC signatures (Fig. S6G).

### Transcriptomic characteristics of myeloid cells in gastric cancer

**Fig. 6:**
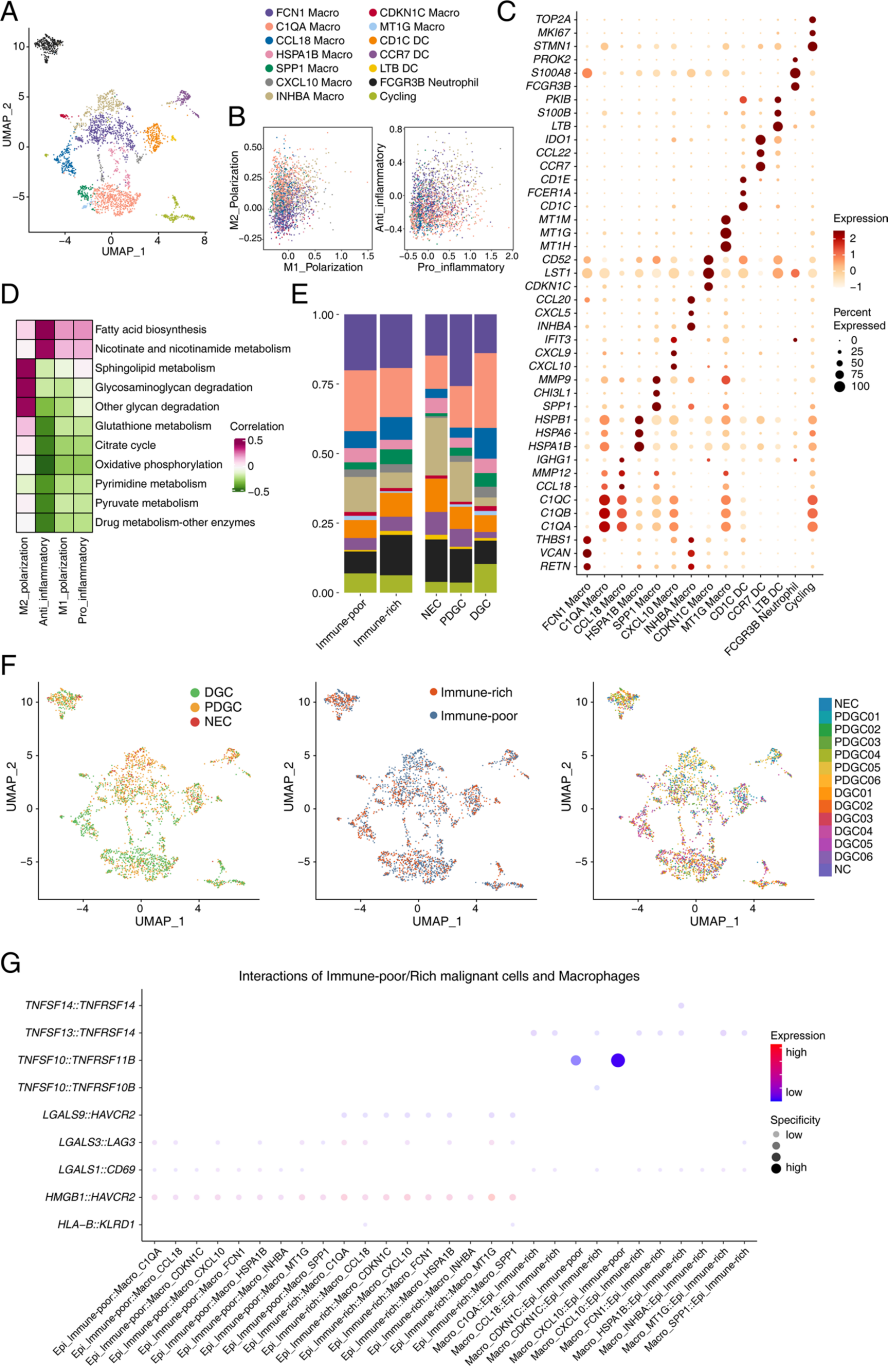
Transcriptomic characteristics of myeloid cells in gastric cancer. **A** UMAP plot showing different clusters of myeloid cells in gastric cancer. **B** The expressing scores of M1 polarization gene set, M2 polarization gene set, pro-inflammatory gene set and anti-inflammatory gene set in myeloid cells. **C** The expression of corresponding markers for different myeloid cell clusters. **D** Correlation of metabolic activity-related gene sets with M1 polarization gene set, M2 polarization gene set, pro-inflammatory gene set and anti-inflammatory gene set in myeloid cells. **E** The percentage of different types of myeloid cells in immune-rich and immune-poor type gastric cancer, and differentiated gastric cancer (DGC), poorly differentiated gastric cancer (PDGC) and neuroendocrine carcinoma (NEC). **F** UMAP plot showing the distribution of myeloid cells across NEC, PDGCs and DGCs, immune-rich and immune-poor types. **G** Interactions between immune-poor/rich type epithelial cells and macrophages inferred from cell communication analysis using the toolkit NATMI

Macrophages account for the main part of myeloid cells (9 identified clusters, with different marker genes such as *FCN1*, *C1QA*, *CCL18*, *HSPA18*, *SPP1*, *CXCL10*, *INHBA*, *CDKN1C* and *MT1G*), and other cell clusters were also identified, such as CD1C^+^ DCs, CCR7^+^ DCs, LTB^+^ DCs, FCGR3B^+^ neutrophils and cycling myeloid cells, according to the corresponding marker genes. Of them, FCN1^+^ macrophages and C1QA^+^ macrophages account for the largest proportion of myeloid cells (Fig. 6A, C). For macrophages, several gene sets, such as the M1 polarization gene set, M2 polarization gene set, pro-inflammatory gene set and anti-inflammatory gene set, were used to score macrophages, and all clusters of macrophages tended to exhibit M2 polarization signatures (Fig. 6B). We identified consistent positive correlation of the above signatures with metabolic activities, such as fatty acid biosynthesis and nicotinate-related metabolism, as well as diverse correlations with energy metabolism, such as the TCA cycle and oxidative phosphorylation (Fig. 6D).

Moreover, we screened the distribution of myeloid cells in diverse classifications, such as DGCs and PDGCs, and immune-rich and immune-poor types. We observed that the proportion of INHBA^+^ macrophages increased in the immune-poor type compared with the immune-rich type and increased in NECs compared with DGCs, which was in accordance with the aforementioned findings and indicated that INHBA^+^ macrophages might influence gastric cancers by repressing the immune responses (Fig. 6E, F). Finally, we identified ligand–receptor interaction pairs of malignant cells and macrophages. For the immune-rich and immune-poor types of malignant cells, we identified the specific interaction *TNFSF10-TNFRSF11B* in the pairs of CDKN1C^+^ macrophages-malignant cells and CXCL10^+^ macrophages-malignant cells, which corresponds to the inhibitory function of TNFRSF11B in immune responses (Fig. 6G).

## Discussion

Our pathology-informed sampling strategy provided exact pathological information on the tumor tissue we sequenced to minimize the influence of the high intra-tumoral heterogeneities, resulting in good discriminations between DGC and PDGC as well as between the immune-poor type and immune-rich type. Additionally, the CNV information inferred from RNA data could also be validated by WES data from corresponding tissue by laser capture microdissection [42]. Taking advantage of this and the single-cell sequencing technology [43], we could clearly distinguish malignant cells from non-malignant epithelial cells by combining clustering and CNV information and achieve a more accurate clustering of tumor cells. By comparing DGC and PDGC, we identified the EMT signature enriched in PDGC, which is consistent with the malignant type associated with the poor differentiation state. More interestingly, our differentiation signatures overlapped with immune infiltration-related genes screened by comparing the immune-rich type with the immune-poor type.

The most interesting finding of our study is the transition of differentiation status in mixed adenoneuroendocrine carcinoma. A pseudotime trajectory of all the malignant cells from DGCs to NECs was constructed, and genes that changed gradually along the trans-differentiation path were identified, indicating a convergence to a small-cell neuroendocrine state reported by Nikolas et al. [44]. *MUC1*, *CEACAM5* and *CLDN18* that are potential targets of immunotherapy were gradually downregulated, indicating the increasing evasion of immune attack during this neuroendocrine differentiation process. The malignant cells of intermediate state showed double-negative signatures for DGC and NEC marker genes, representing a possible “stem/progenitor cell” signature that might be associated with gastric cancer progression. Similar to studies in prostate cancer and non-small-cell lung cancer, many transcription factors were found to be differentially expressed in this trans-differentiation path [45, 46]. The new finding is the negative correlation of interferon-related pathways found along the DGC to NEC trans-differentiation path, which was also accompanied by reduced CD8^+^ T lymph cell infiltration in IHC staining, supporting neuroendocrine differentiation as a mechanism for immune evasion in gastric cancer, which needs further functional investigations in the future.

## Supplementary Information

Below is the link to the electronic supplementary material.

Supplementary file1 (PDF 10673 KB)

Supplementary file2 (XLSX 417 KB)

## Data Availability

The raw sequence data reported in this paper have been deposited in the Genome Sequence Archive in National Genomics Data Center (National Genomics Data Center Members and Partners, 2020), Beijing Institute of Genomics (China National Center for Bioinformation), Chinese Academy of Sciences, under accession number HRA002108 that are publicly accessible at http://bigd.big.ac.cn/gsa-human.
