# Supplementary material for: Relationships of tumor differentiation and immune infiltration in gastric cancers revealed by single-cell RNA-seq analyses
Source: Cell Mol Life Sci. 2023 Feb 2;80(2):57. doi: 10.1007/s00018-023-04702-1 (PMC9894979; doi:10.1007/s00018-023-04702-1)
Supplement: Supplementary file 1 — Supplementary file1 (PDF 10673 KB) [file 18_2023_4702_MOESM1_ESM.pdf]

Fig S1

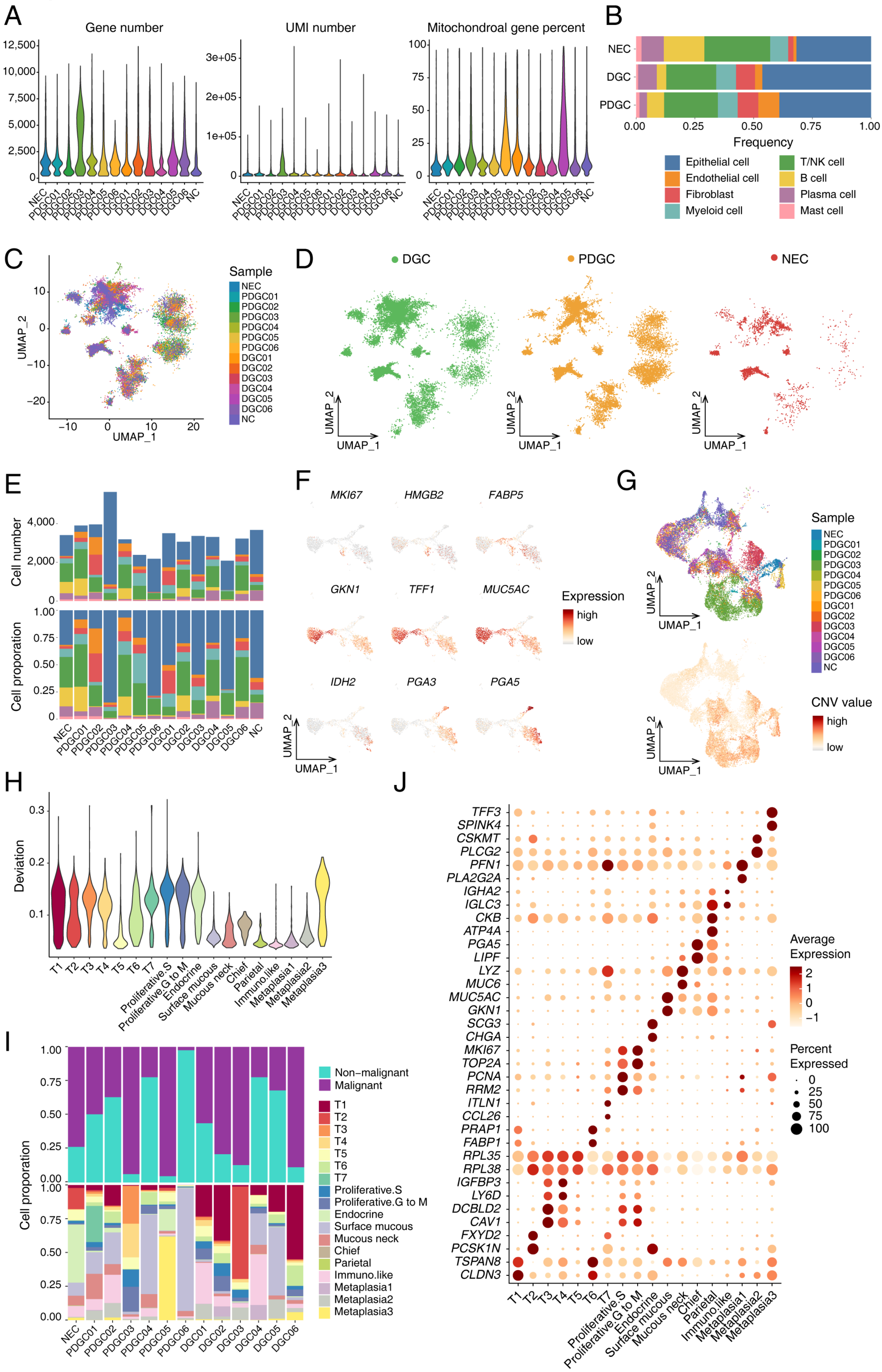
A
Gene number
UMI number
Mitochondroal gene percent
B
NEC
DGC
PDGC
Frequency
Epithelial cell
Endothelial cell
Fibroblast
Myeloid cell
T/NK cell
B cell
Plasma cell
Mast cell
C
Sample
UMAP_1
UMAP_2
D
DGC
PDGC
NEC
E
Cell number
Cell proporation
F
MKI67
HMGB2
FABP5
GKN1
TFF1
MUC5AC
IDH2
PGA3
PGA5
Expression
high
low
G
Sample
CNV value
high
low
H
Deviation
I
Cell proporation
Non−malignant
Malignant
T1
T2
T3
T4
T5
T6
T7
Proliferative.S
Proliferative.G to M
Endocrine
Surface mucous
Mucous neck
Chief
Parietal
Immuno.like
Metaplasia1
Metaplasia2
Metaplasia3
J
Average Expression
Percent Expressed
TFF3
SPINK4
CSKMT
PLCG2
PFN1
PLA2G2A
IGHA2
IGLC3
CKB
ATP4A
PGA5
LIPF
LYZ
MUC6
MUC5AC
GKN1
SCG3
CHGA
MKI67
TOP2A
PCNA
RRM2
ITLN1
CCL26
PRAP1
FABP1
RPL35
RPL38
IGFBP3
LY6D
DCBLD2
CAV1
FXYD2
PCSK1N
TSPAN8
CLDN3

Figure S1.

Representative single-cell transcriptome landscape of gastric cancer. **A** Violin plot showing detected gene number, transcripts number and mitochondrial gene percentage of each sample. **B** The distribution of all cell types in differentiated gastric cancer (DGC), poorly differentiated gastric cancer (PDGC) and neuroendocrine carcinoma (NEC). **C** Different sample sources information shown in UMAP plot (n = 46,883). **D** UMAP plot showing the distribution of cells from DGC, PDGC and NEC. **E** The cell number and proportion of different cell types in each sample. **F** UMAP plot showing the expression of different cell type markers in the normal mucosa tissue sample. **G** UMAP plot showing sample sources and CNV deviation information. **H** Violin plot showing CNV deviation of different epithelial cell clusters from non-malignant and malignant epithelial cells. **I** The percentage of malignant cells and different epithelial cell clusters in each sample. **J** The expression of corresponding markers for different epithelial cell clusters.

Fig S2

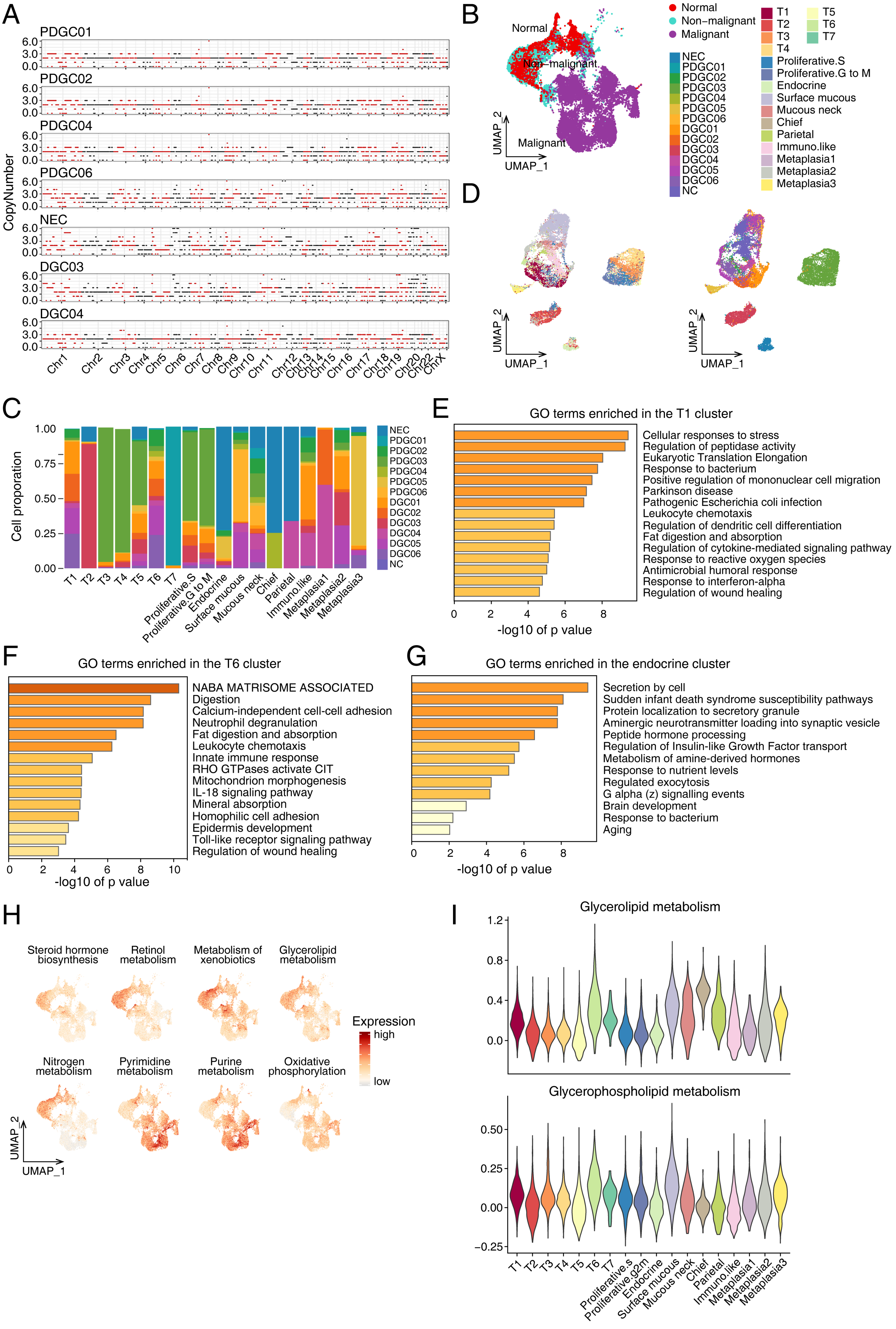
A
PDGC01
PDGC02
PDGC04
PDGC06
NEC
DGC03
DGC04
CopyNumber
6.0
3.0
0.0
Chr1 Chr2 Chr3 Chr4 Chr5 Chr6 Chr7 Chr8 Chr9 Chr10 Chr11 Chr12 Chr13 Chr14 Chr15 Chr16 Chr17 Chr18 Chr19 Chr20 Chr22 ChrX
B
Normal
Non−malignant
Malignant
UMAP_2
UMAP_1
Normal
Non−malignant
Malignant
NEC
PDGC01
PDGC02
PDGC03
PDGC04
PDGC05
PDGC06
DGC01
DGC02
DGC03
DGC04
DGC05
DGC06
NC
T1
T2
T3
T4
T5
T6
T7
Proliferative.S
Proliferative.G to M
Endocrine
Surface mucous
Mucous neck
Chief
Parietal
Immuno.like
Metaplasia1
Metaplasia2
Metaplasia3
D
UMAP_2
UMAP_1
C
Cell proporation
1.00
0.75
0.50
0.25
0.00
T1 T2 T3 T4 T5 T6 T7 Proliferative.S Proliferative.G to M Endocrine Surface mucous Mucous neck Chief Parietal Immuno.like Metaplasia1 Metaplasia2 Metaplasia3
E
GO terms enriched in the T1 cluster
Cellular responses to stress
Regulation of peptidase activity
Eukaryotic Translation Elongation
Response to bacterium
Positive regulation of mononuclear cell migration
Parkinson disease
Pathogenic Escherichia coli infection
Leukocyte chemotaxis
Regulation of dendritic cell differentiation
Fat digestion and absorption
Regulation of cytokine-mediated signaling pathway
Response to reactive oxygen species
Antimicrobial humoral response
Response to interferon-alpha
Regulation of wound healing
-log10 of p value
F
GO terms enriched in the T6 cluster
NABA MATRISOME ASSOCIATED
Digestion
Calcium-independent cell-cell adhesion
Neutrophil degranulation
Fat digestion and absorption
Leukocyte chemotaxis
Innate immune response
RHO GTPases activate CIT
Mitochondrion morphogenesis
IL-18 signaling pathway
Mineral absorption
Homophilic cell adhesion
Epidermis development
Toll-like receptor signaling pathway
Regulation of wound healing
-log10 of p value
G
GO terms enriched in the endocrine cluster
Secretion by cell
Sudden infant death syndrome susceptibility pathways
Protein localization to secretory granule
Aminergic neurotransmitter loading into synaptic vesicle
Peptide hormone processing
Regulation of Insulin-like Growth Factor transport
Metabolism of amine-derived hormones
Response to nutrient levels
Regulated exocytosis
G alpha (z) signalling events
Brain development
Response to bacterium
Aging
-log10 of p value
H
Steroid hormone biosynthesis
Retinol metabolism
Metabolism of xenobiotics
Glycerolipid metabolism
Nitrogen metabolism
Pyrimidine metabolism
Purine metabolism
Oxidative phosphorylation
Expression
high
low
UMAP_2
UMAP_1
I
Glycerolipid metabolism
Glycerophospholipid metabolism
T1 T2 T3 T4 T5 T6 T7 Proliferative.s Proliferative.g2m Endocrine Surface mucous Mucous neck Chief Parietal Immuno.like Metaplasia1 Metaplasia2 Metaplasia3

Figure S2.

Representative single-cell transcriptome landscape of gastric cancer. **A** Chromosomal landscape of large-scale copy number variations (CNV) inferred from whole exome sequencing data of the corresponding laser capture microdissected tissue. **B** UMAP plot showing normal, non-malignant and malignant epithelial cell clusters. **C** The percentage of cells from different sample in each cluster. D UMAP plot showing different epithelial cell clusters according to gene regulatory networks (GRNs). **E** Gene enrichment analysis showing the signaling pathways relatively up-regulated in T1 clusters. **F** Gene enrichment analysis showing the signaling pathways relatively up-regulated in T6 clusters. **G** Gene enrichment analysis showing the signaling pathways relatively up-regulated in endocrine clusters. **H** UMAP plot showing the metabolism-related activities in epithelial clusters. **I** Violin plot showing the expression levels of gene set signatures including glycerolipid metabolism and glycerophospholipid metabolism in different epithelial cell clusters.

Fig S3

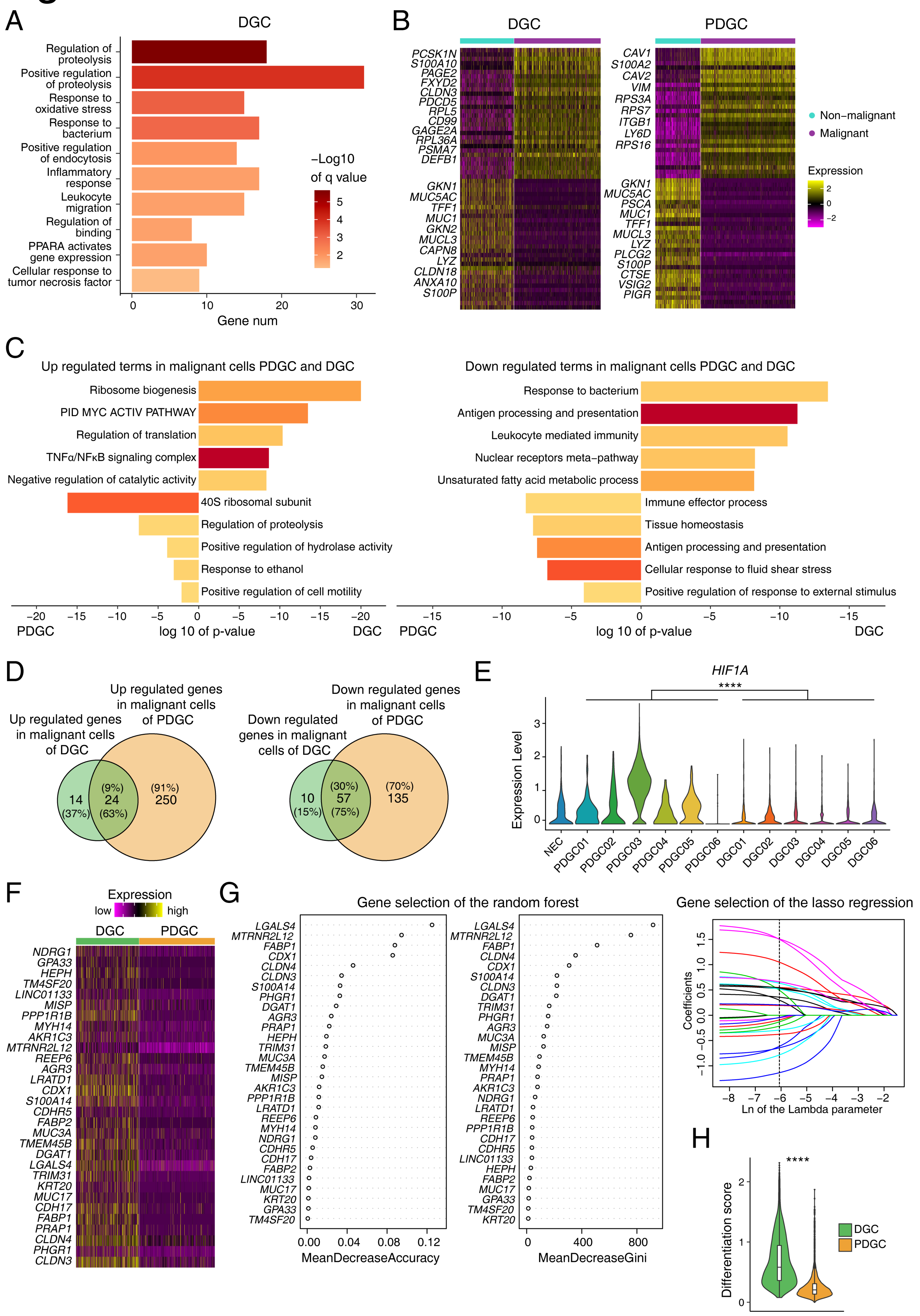

A
DGC
Regulation of proteolysis
Positive regulation of proteolysis
Response to oxidative stress
Response to bacterium
Positive regulation of endocytosis
Inflammatory response
Leukocyte migration
Regulation of binding
PPARA activates gene expression
Cellular response to tumor necrosis factor
−Log10 of q value
5
4
3
2
0
10
20
30
Gene num
B
DGC
PDGC
PCSK1N
S100A10
PAGE2
FXYD2
CLDN3
PDCD5
RPL5
CD99
GAGE2A
RPL36A
PSMA7
DEFB1
GKN1
MUC5AC
TFF1
MUC1
GKN2
MUCL3
CAPN8
LYZ
CLDN18
ANXA10
S100P
CAV1
S100A2
CAV2
VIM
RPS3A
RPS7
ITGB1
LY6D
RPS16
GKN1
MUC5AC
PSCA
MUC1
TFF1
MUCL3
LYZ
PLCG2
S100P
CTSE
VSIG2
PIGR
Non−malignant
Malignant
Expression
2
0
−2
C
Up regulated terms in malignant cells PDGC and DGC
Ribosome biogenesis
PID MYC ACTIV PATHWAY
Regulation of translation
TNFα/NFκB signaling complex
Negative regulation of catalytic activity
40S ribosomal subunit
Regulation of proteolysis
Positive regulation of hydrolase activity
Response to ethanol
Positive regulation of cell motility
−20
−15
−10
−5
0
−5
−10
−15
−20
PDGC
log 10 of p-value
DGC
Down regulated terms in malignant cells PDGC and DGC
Response to bacterium
Antigen processing and presentation
Leukocyte mediated immunity
Nuclear receptors meta−pathway
Unsaturated fatty acid metabolic process
Immune effector process
Tissue homeostasis
Antigen processing and presentation
Cellular response to fluid shear stress
Positive regulation of response to external stimulus
−15
−10
−5
0
−5
−10
−15
PDGC
log 10 of p-value
DGC
D
Up regulated genes in malignant cells of DGC
Up regulated genes in malignant cells of PDGC
14 (37%)
(9%) 24 (63%)
(91%) 250
Down regulated genes in malignant cells of DGC
Down regulated genes in malignant cells of PDGC
10 (15%)
(30%) 57 (75%)
(70%) 135
E
HIF1A
****
Expression Level
3
2
1
0
NEC
PDGC01
PDGC02
PDGC03
PDGC04
PDGC05
PDGC06
DGC01
DGC02
DGC03
DGC04
DGC05
DGC06
F
Expression
low
high
DGC
PDGC
NDRG1
GPA33
HEPH
TM4SF20
LINC01133
MISP
PPP1R1B
MYH14
AKR1C3
MTRNR2L12
REEP6
AGR3
LRATD1
CDX1
S100A14
CDHR5
FABP2
MUC3A
TMEM45B
DGAT1
LGALS4
TRIM31
KRT20
MUC17
CDH17
FABP1
PRAP1
CLDN4
PHGR1
CLDN3
G
Gene selection of the random forest
LGALS4
MTRNR2L12
FABP1
CDX1
CLDN4
CLDN3
S100A14
PHGR1
DGAT1
AGR3
PRAP1
HEPH
TRIM31
MUC3A
TMEM45B
MISP
AKR1C3
PPP1R1B
LRATD1
REEP6
MYH14
NDRG1
CDHR5
CDH17
FABP2
LINC01133
MUC17
KRT20
GPA33
TM4SF20
0.00
0.04
0.08
0.12
MeanDecreaseAccuracy
LGALS4
MTRNR2L12
FABP1
CLDN4
CDX1
S100A14
CLDN3
DGAT1
TRIM31
PHGR1
AGR3
MUC3A
MISP
TMEM45B
MYH14
PRAP1
AKR1C3
NDRG1
LRATD1
REEP6
PPP1R1B
CDH17
CDHR5
LINC01133
HEPH
FABP2
MUC17
GPA33
TM4SF20
KRT20
0
400
800
MeanDecreaseGini
Gene selection of the lasso regression
Coefficients
1.5
1.0
0.5
0.0
−0.5
−1.0
−8
−7
−6
−5
−4
−3
−2
Ln of the Lambda parameter
H
****
Differentiation score
2
1
0
DGC
PDGC

Figure S3.

Comparison between differentiated gastric cancer (DGC) and poorly differentiated gastric cancer (PDGC). **A** Gene enrichment analysis showing the functions relatively up-regulated in DGC. **B** Heatmap showing differentially expressed genes (DEG) of PDGC and DGC compared with non-malignant epithelial cells. Part gene names are displayed. **C** Gene enrichment analysis showing the signaling pathways relatively up- or down-regulated in PDGC and DGC compared with non-malignant epithelial cells. **D** Venn diagram showing the number of up- and down-regulated genes in PDGC and DGC compared with non-malignant epithelial cells. **E** Violin plot showing the expression levels of *HIF1A* in different samples (**** - p value < 0.0001 calculated by wilcox test). **F** Heatmap showing the expression levels of genes which have the high correlation with DEGs of DGC compared to PDGC. **G** The processing of gene selection using the random forest and lasso regression methods. The left point plot showing the contributions of genes to the predicting output of DGC and PDGC. The right line plot showing the choices of the lambda values of the lasso regression. **H** Violin plot showing differentiation score in DGC and PDGC (**** - p value < 0.0001 calculated by wilcox test).

Fig S4

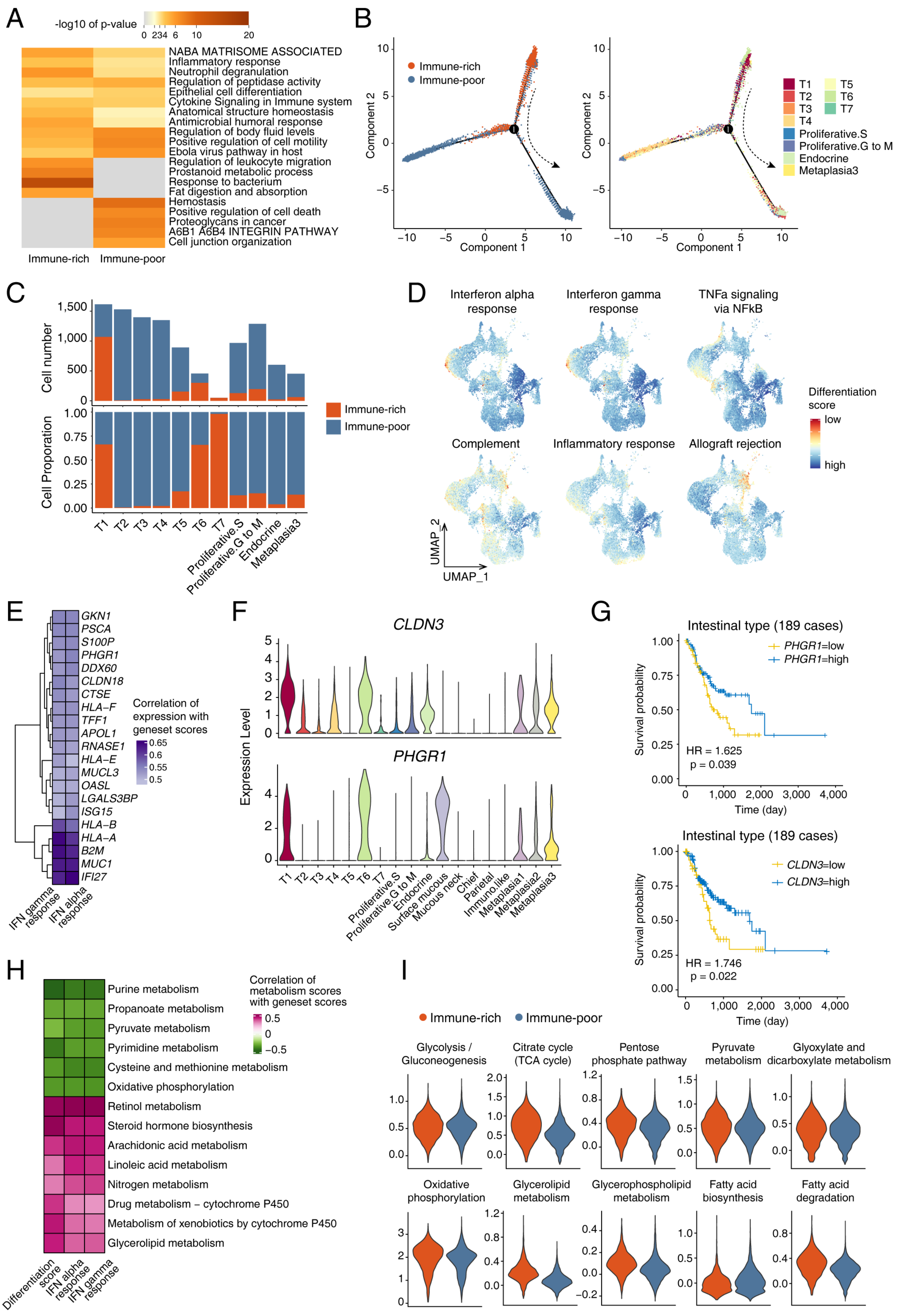

A
-log10 of p-value
0 2 3 4 6 10 20
NABA MATRISOME ASSOCIATED
Inflammatory response
Neutrophil degranulation
Regulation of peptidase activity
Epithelial cell differentiation
Cytokine Signaling in Immune system
Anatomical structure homeostasis
Antimicrobial humoral response
Regulation of body fluid levels
Positive regulation of cell motility
Ebola virus pathway in host
Regulation of leukocyte migration
Prostanoid metabolic process
Response to bacterium
Fat digestion and absorption
Hemostasis
Positive regulation of cell death
Proteoglycans in cancer
A6B1 A6B4 INTEGRIN PATHWAY
Cell junction organization
Immune-rich
Immune-poor
B
Immune-rich
Immune-poor
Component 2
Component 1
T1
T2
T3
T4
T5
T6
T7
Proliferative.S
Proliferative.G to M
Endocrine
Metaplasia3
C
Cell number
Cell Proporation
Immune-rich
Immune-poor
T1
T2
T3
T4
T5
T6
T7
Proliferative.S
Proliferative.G to M
Endocrine
Metaplasia3
D
Interferon alpha response
Interferon gamma response
TNFa signaling via NFkB
Complement
Inflammatory response
Allograft rejection
Differentiation score
low
high
UMAP_2
UMAP_1
E
GKN1
PSCA
S100P
PHGR1
DDX60
CLDN18
CTSE
HLA−F
TFF1
APOL1
RNASE1
HLA−E
MUCL3
OASL
LGALS3BP
ISG15
HLA−B
HLA−A
B2M
MUC1
IFI27
Correlation of expression with geneset scores
0.65
0.6
0.55
0.5
IFN gamma response
IFN alpha response
F
CLDN3
PHGR1
Expression Level
T1
T2
T3
T4
T5
T6
T7
Proliferative.S
Proliferative.G to M
Endocrine
Surface mucous
Mucous neck
Chief
Parietal
Immuno.like
Metaplasia1
Metaplasia2
Metaplasia3
G
Intestinal type (189 cases)
PHGR1=low
PHGR1=high
Survival probability
HR = 1.625
p = 0.039
Time (day)
Intestinal type (189 cases)
CLDN3=low
CLDN3=high
Survival probability
HR = 1.746
p = 0.022
Time (day)
H
Purine metabolism
Propanoate metabolism
Pyruvate metabolism
Pyrimidine metabolism
Cysteine and methionine metabolism
Oxidative phosphorylation
Retinol metabolism
Steroid hormone biosynthesis
Arachidonic acid metabolism
Linoleic acid metabolism
Nitrogen metabolism
Drug metabolism – cytochrome P450
Metabolism of xenobiotics by cytochrome P450
Glycerolipid metabolism
Correlation of metabolism scores with geneset scores
0.5
0
−0.5
Differentiation score
IFN alpha response
IFN gamma response
I
Immune-rich
Immune-poor
Glycolysis / Gluconeogenesis
Citrate cycle (TCA cycle)
Pentose phosphate pathway
Pyruvate metabolism
Glyoxylate and dicarboxylate metabolism
Oxidative phosphorylation
Glycerolipid metabolism
Glycerophospholipid metabolism
Fatty acid biosynthesis
Fatty acid degradation

Figure S4.

Transcriptomic profiling of immune infiltration-related genes. **A** Gene enrichment analysis showing the signaling pathways relatively up- or down-regulated in immune-rich and immune-poor type gastric cancer. **B** Pseudotime trajectory of malignant epithelial cells according to the immune-related gene sets. **C** Number and percentage of immune-rich and immune-poor type cells in each malignant epithelial cluster. **D** UMAP plot showing expression of hallmark signatures and GO gene sets of immune response in epithelial cells. **E** Genes highly correlated with expression of interferon gamma and the interferon alpha pathways. **F** Violin plot showing expression levels of *PHGR1* and *CLDN3* in each epithelial cell cluster. **G** Kaplan-Meier survival curve showing the survival of intestinal-type gastric cancer cases in the TCGA dataset with high and low expression levels of *PHGR1* and *CLDN3*. **H** Metabolic activity-related gene sets highly correlated with expression of the differentiation gene set and interferon alpha/gamma response gene sets. **I** Violin plot showing expression levels metabolic activity-related gene sets in immune-rich and immune-poor type gastric cancer.

Fig S5

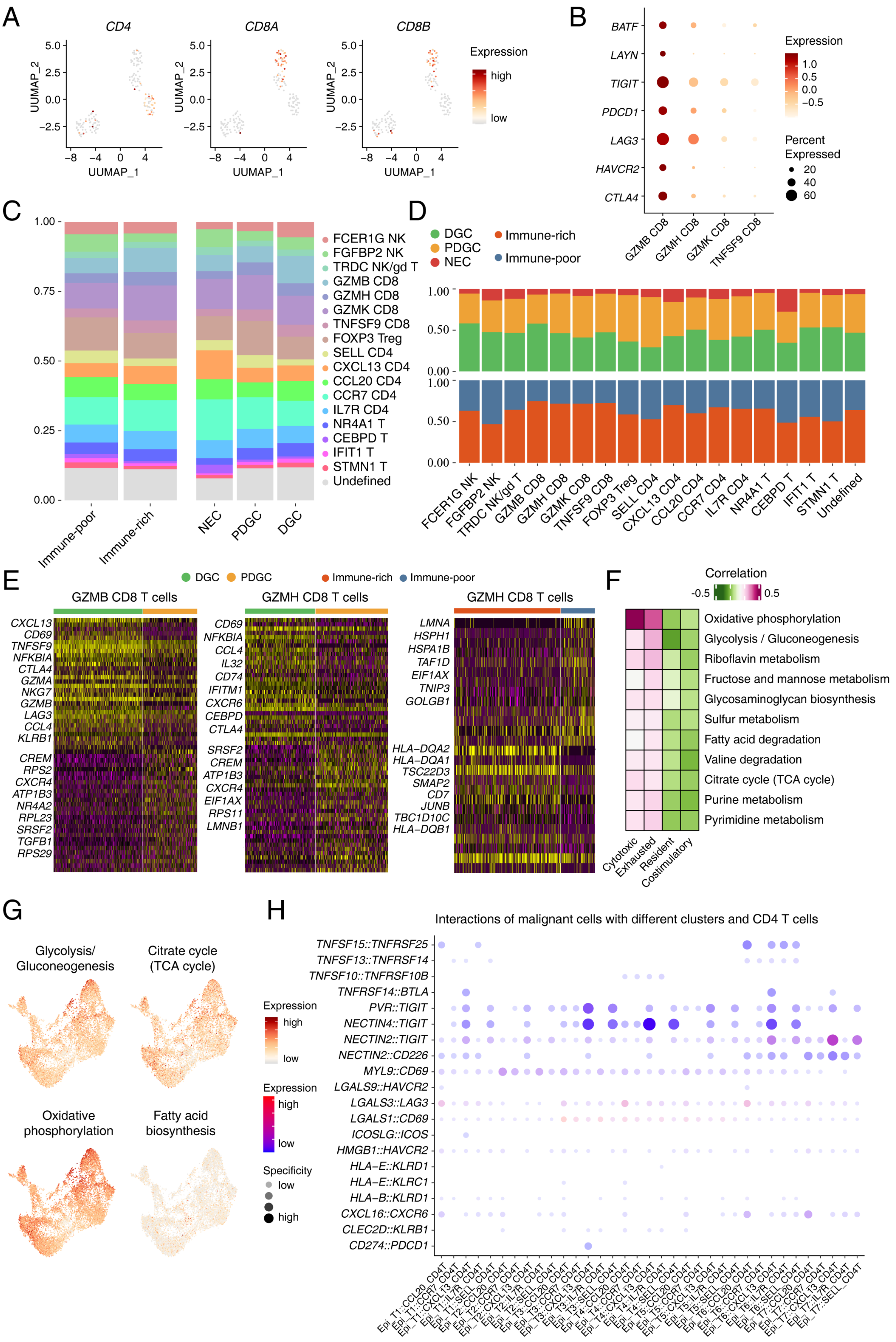

A
CD4
CD8A
CD8B
UUMAP_2
UUMAP_1
Expression
high
low
B
BATF
LAYN
TIGIT
PDCD1
LAG3
HAVCR2
CTLA4
GZMB CD8
GZMH CD8
GZMK CD8
TNFSF9 CD8
Expression
Percent Expressed
C
Immune-poor
Immune-rich
NEC
PDGC
DGC
FCER1G NK
FGFBP2 NK
TRDC NK/gd T
GZMB CD8
GZMH CD8
GZMK CD8
TNFSF9 CD8
FOXP3 Treg
SELL CD4
CXCL13 CD4
CCL20 CD4
CCR7 CD4
IL7R CD4
NR4A1 T
CEBPD T
IFIT1 T
STMN1 T
Undefined
D
DGC
PDGC
NEC
Immune-rich
Immune-poor
E
DGC
PDGC
Immune-rich
Immune-poor
GZMB CD8 T cells
GZMH CD8 T cells
GZMH CD8 T cells
CXCL13
CD69
TNFSF9
NFKBIA
CTLA4
GZMA
NKG7
GZMB
LAG3
CCL4
KLRB1
CREM
RPS2
CXCR4
ATP1B3
NR4A2
RPL23
SRSF2
TGFB1
RPS29
CD69
NFKBIA
CCL4
IL32
CD74
IFITM1
CXCR6
CEBPD
CTLA4
SRSF2
CREM
ATP1B3
CXCR4
EIF1AX
RPS11
LMNB1
LMNA
HSPH1
HSPA1B
TAF1D
EIF1AX
TNIP3
GOLGB1
HLA−DQA2
HLA−DQA1
TSC22D3
SMAP2
CD7
JUNB
TBC1D10C
HLA−DQB1
F
Correlation
Oxidative phosphorylation
Glycolysis / Gluconeogenesis
Riboflavin metabolism
Fructose and mannose metabolism
Glycosaminoglycan biosynthesis
Sulfur metabolism
Fatty acid degradation
Valine degradation
Citrate cycle (TCA cycle)
Purine metabolism
Pyrimidine metabolism
Cytotoxic
Exhausted
Resident
Costimulatory
G
Glycolysis/ Gluconeogenesis
Citrate cycle (TCA cycle)
Oxidative phosphorylation
Fatty acid biosynthesis
H
Interactions of malignant cells with different clusters and CD4 T cells
Expression
high
low
Expression
high
low
Specificity
low
high
TNFSF15::TNFRSF25
TNFSF13::TNFRSF14
TNFSF10::TNFRSF10B
TNFRSF14::BTLA
PVR::TIGIT
NECTIN4::TIGIT
NECTIN2::TIGIT
NECTIN2::CD226
MYL9::CD69
LGALS9::HAVCR2
LGALS3::LAG3
LGALS1::CD69
ICOSLG::ICOS
HMGB1::HAVCR2
HLA−E::KLRD1
HLA−E::KLRC1
HLA−B::KLRD1
CXCL16::CXCR6
CLEC2D::KLRB1
CD274::PDCD1

Figure S5.

Tumor infiltrating T cell characteristics and interactions with malignant cells. **A** UMAP plot showing expression of *CD4*, *CD8A* and *CD8B* in *STMN1*$^{+}$ T cells. **B** The expression of exhaustion-related genes in different types of CD8^{+} T cells. **C** The percentage of different types of T cells in immune-rich and immune-poor type gastric cancer, and differentiated gastric cancer (DGC), poorly differentiated gastric cancer (PDGC) and neuroendocrine carcinoma (NEC). **D** The percentage of T cells from NEC, PDGC and NEC, and immune-rich and immune-poor type in different T cell clusters. **E** Heatmap showing differentially expressed genes between *GZMH*$^{+}$ CD8^{+} T cells from immune-rich/poor type gastric cancer and DGC/PDGC, and between *GZMB*$^{+}$ CD8^{+} T cells from DGC/PDGC. Part gene names are displayed. **F** Metabolic activity-related gene sets highly correlated with cytotoxic, exhausted, resident, costimulatory and cell cycle signatures of CD8^{+} T cells. **G** UMAP plot showing expression of metabolic activity-related gene sets in T cells. **H** Interactions between epithelial cells and CD4+ T cells inferred from cell communication analysis using toolkit NATMI.

Fig S6

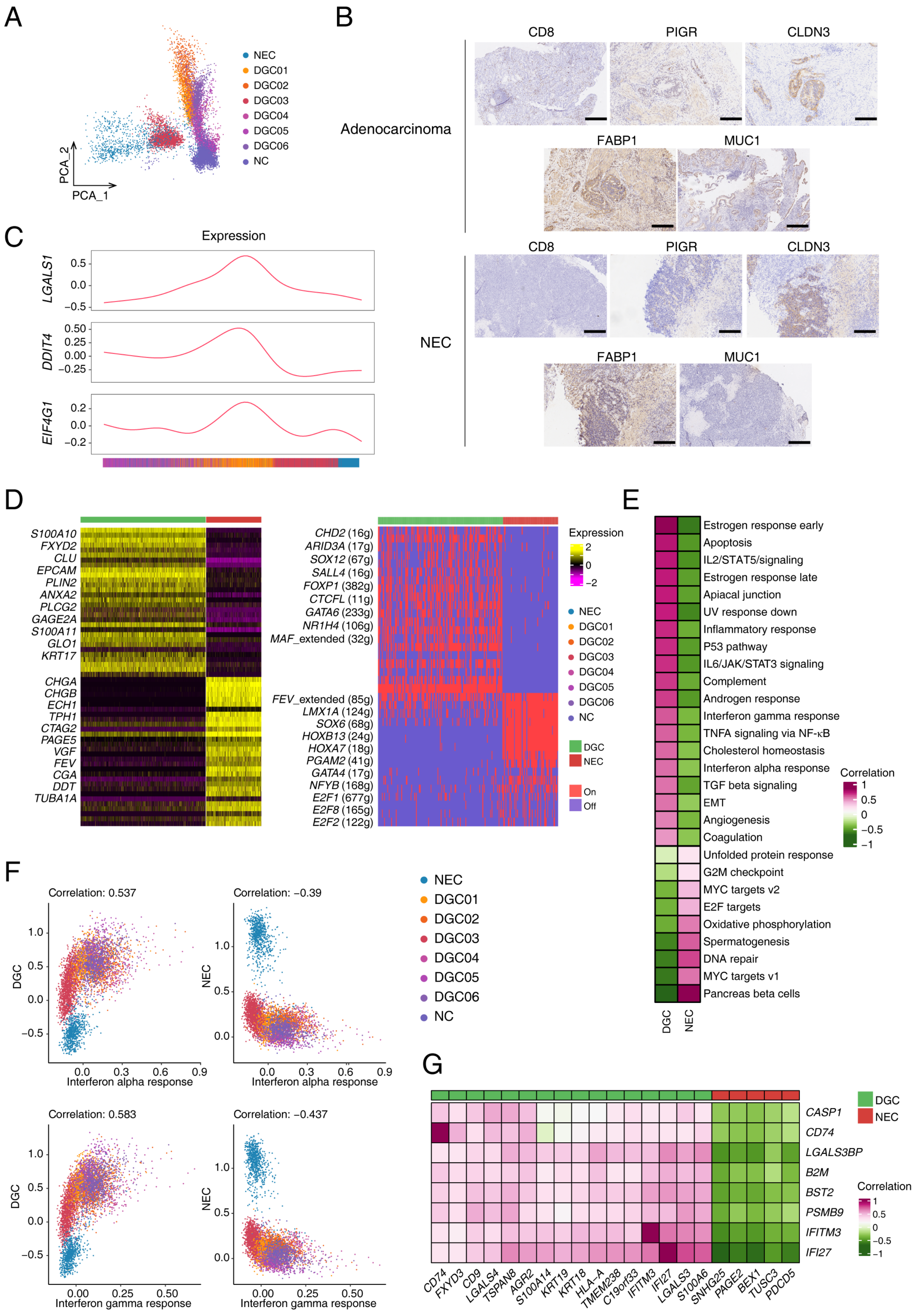

A
NEC
DGC01
DGC02
DGC03
DGC04
DGC05
DGC06
NC
PCA_2
PCA_1
B
Adenocarcinoma
CD8
PIGR
CLDN3
FABP1
MUC1
NEC
CD8
PIGR
CLDN3
FABP1
MUC1
C
Expression
LGALS1
0.5
0.0
−0.5
DDIT4
0.50
0.25
0.00
−0.25
EIF4G1
0.2
0.0
−0.2
D
S100A10
FXYD2
CLU
EPCAM
PLIN2
ANXA2
PLCG2
GAGE2A
S100A11
GLO1
KRT17
CHGA
CHGB
ECH1
TPH1
CTAG2
PAGE5
VGF
FEV
CGA
DDT
TUBA1A
CHD2 (16g)
ARID3A (17g)
SOX12 (67g)
SALL4 (16g)
FOXP1 (382g)
CTCFL (11g)
GATA6 (233g)
NR1H4 (106g)
MAF_extended (32g)
FEV_extended (85g)
LMX1A (124g)
SOX6 (68g)
HOXB13 (24g)
HOXA7 (18g)
PGAM2 (41g)
GATA4 (17g)
NFYB (168g)
E2F1 (677g)
E2F8 (165g)
E2F2 (122g)
Expression
2
0
−2
NEC
DGC01
DGC02
DGC03
DGC04
DGC05
DGC06
NC
DGC
NEC
On
Off
E
Estrogen response early
Apoptosis
IL2/STAT5/signaling
Estrogen response late
Apiacal junction
UV response down
Inflammatory response
P53 pathway
IL6/JAK/STAT3 signaling
Complement
Androgen response
Interferon gamma response
TNFA signaling via NF-κB
Cholesterol homeostasis
Interferon alpha response
TGF beta signaling
EMT
Angiogenesis
Coagulation
Unfolded protein response
G2M checkpoint
MYC targets v2
E2F targets
Oxidative phosphorylation
Spermatogenesis
DNA repair
MYC targets v1
Pancreas beta cells
DGC
NEC
Correlation
1
0.5
0
−0.5
−1
F
Correlation: 0.537
DGC
1.0
0.5
0.0
−0.5
0.0
0.3
0.6
0.9
Interferon alpha response
Correlation: −0.39
NEC
1.0
0.5
0.0
0.0
0.3
0.6
0.9
Interferon alpha response
NEC
DGC01
DGC02
DGC03
DGC04
DGC05
DGC06
NC
Correlation: 0.583
DGC
1.0
0.5
0.0
−0.5
0.00
0.25
0.50
Interferon gamma response
Correlation: −0.437
NEC
1.0
0.5
0.0
0.00
0.25
0.50
Interferon gamma response
G
CASP1
CD74
LGALS3BP
B2M
BST2
PSMB9
IFITM3
IFI27
DGC
NEC
Correlation
1
0.5
0
−0.5
−1
CD74
FXYD3
CD9
LGALS4
TSPAN8
AGR2
S100A14
KRT19
KRT18
HLA-A
TMEM238
C19orf33
IFITM3
IFI27
LGALS3
S100A6
SNHG25
PAGE2
BEX1
TUSC3
PDCD5

Figure S6.

Transition of differentiation status in mixed adenoneuroendocrine carcinoma. **A** PCA plot showing the distribution of epithelial cells from differentiated gastric adenocarcinoma (DGC), intermediate state (DGC01 and DGC03) and neuroendocrine carcinoma (NEC). **B** Representative images of immune-histochemical staining of CD8 and other differentially expressed proteins in adenocarcinoma and NEC components from the same mixed adenoneuroendocrine carcinoma. Scale bar, 300 μm. **C** Representative genes that enriched in the intermediate state. **D** Heatmap showing differentially expressed genes between DGC and NEC and gene regulatory networks that changed gradually along the trans-differentiation path of DGC to NEC. Part gene and TF names are displayed. **E** Hallmark gene signatures highly correlated with DGC and NEC. **F** The correlation between interferon alpha/gamma response genes and DGC, intermediate state (DGC01 and DGC03) and NEC. **G** The correlation between specific genes in interferon-related pathways and DGC/NEC.
